# Correlating Anatomy and Function with Gene Expression in Individual Neurons by Combining *in Vivo* Labeling, Patch Clamp, and Single Cell RNA-seq

**DOI:** 10.3389/fncel.2017.00376

**Published:** 2017-11-30

**Authors:** Carsten K. Pfeffer, Riccardo Beltramo

**Affiliations:** ^1^Neurobiology Section, Center for Neural Circuits and Behavior, University of California, San Diego, La Jolla, CA, United States; ^2^Department of Neuroscience, University of California, San Diego, La Jolla, CA, United States

**Keywords:** single cell transcriptomics, patch-clamp, *in vivo* imaging, tracing experiments, visual cortex

## Abstract

The classification of neurons into distinct types is an ongoing effort aimed at revealing and understanding the diversity of the components of the nervous system. Recently available methods allow us to determine the gene expression pattern of individual neurons in the mammalian cerebral cortex to generate powerful categorization schemes. For a thorough understanding of neuronal diversity such genetic categorization schemes need to be combined with traditional classification parameters like position, axonal projection or response properties to sensory stimulation. Here we describe a method to link the gene expression of individual neurons with their position, axonal projection, or sensory response properties. Neurons are labeled *in vivo* based on their anatomical or functional properties and, using patch clamp pipettes, their RNA individually harvested *in vitro* for RNAseq. We validate the methodology using multiple established molecularly and anatomically distinct cell populations and explore molecular differences between uncharacterized neurons in mouse visual cortex. Gene expression patterns between L5 neurons projecting to frontal or contralateral cortex are distinct while L2 neurons differing in position, projection, or function are molecularly similar. With this method we can determine the genetic expression pattern of functionally and anatomically identified individual neurons.

## Introduction

The classification of neurons into distinct cell-types is an ongoing effort that began in the nineteenth century (Ramón y Cajal, 1995). Contemporary classification of neurons is based on anatomical parameters, (e.g., where the cell body is located), morphological parameters (e.g., where the neurites project), molecular properties (e.g., what proteins are expressed or transmitters released), and functional properties (e.g., what conditions are necessary for their activation; Ascoli et al., 2008; Defelipe et al., 2013; Fishell and Heintz, 2013). The development of highly efficient nucleic acid sequencing techniques allows us today to determine the gene expression pattern of individual neurons to reveal their molecular identity with unprecedented resolution (Heiman et al., 2008; Tang et al., 2009; Macosko et al., 2015; Zeisel et al., 2015; Tasic et al., 2016). However, matching the transcriptional identity of individual neurons with their anatomical, morphological, or functional properties has been challenging.

Current methods for obtaining single cell transcriptomes are predominantly based on bulk digestion of neural tissue followed by isolation and eventually FAC sorting of single cells (Macosko et al., 2015; Zeisel et al., 2015; Tasic et al., 2016). However, the anatomical and functional identity of individual neurons depends on their specific integration into fine scale circuits within the nervous system. Bulk isolation methods cannot be easily combined with precise positional information about individual neurons. Furthermore, these methods are also not suitable to determine the gene expression pattern of individual neurons in combination with information relative to their activity pattern observed *in vivo*, for example their specific response to sensory stimulation. Thus, approaches need to be developed where the precise positional, anatomical, and functional identity of individual neurons can be combined with their transcriptional profile.

Here we develop an approach that allows us to label neurons *in vivo* according to their position, axonal projection and response properties to sensory stimulation, and individually harvest their RNA *in vitro* for transcriptional profiling by visually targeting these neurons with patch clamp pipettes. Our approach thus significantly extends the applications of a recently reported approach for transcriptome analysis of patched neurons (Cadwell et al., 2016; Fuzik et al., 2016). Furthermore we comprehensively verify and validate our approach on a large number of distinct GABAergic and glutamatergic cell classes whose transcriptome had previously been established through bulk isolation methods (Zeisel et al., 2015; Cembrowski et al., 2016a; Tasic et al., 2016). Finally, we explore and compare the transcriptomes of uncharacterized neuron populations in visual cortex demonstrating that L5 neurons projecting to frontal or contralateral cortex are molecularly distinct while gene expression patterns of L2 neurons differing in their position, projection, or function are similar. The approaches developed and applied here will be essential in understanding the relationship between gene expression of individual neurons and their specific integration into a cortical circuit.

## Results

Our goal is to develop a simple and reliable method to combine transcriptome analysis with physiological and anatomical features of single neurons. We first describe our RNA harvesting approach using patch clamp pipettes and quantify and validate the obtained single neuron transcriptomes by harvesting RNA from established, molecularly distinct classes of neurons. We then describe an approach to determine the gene expression pattern of individual neurons with specific locations, axonal projection patterns and responses to sensory stimulation determined *in vivo*.

### RNA-seq of individually patched neurons: detection threshold, contamination, and variability

RT-PCR of patch-c lamped neurons can be used to identify select genes in individual cells (Lambolez et al., 1992) which we have previously applied to molecularly separate electrophysiologically characterized GABAergic neurons using up to 30 genes (Pfeffer et al., 2013). Here we extend the methodology to determine the single-cell transcriptome through RNA sequencing. Neurons were patched in acute slices of visual cortex with standard patch-clamp pipettes and the cell content, including the nucleus, was harvested into the patch-pipette and subsequently expelled into a single tube (see section Methods). We then adapted the cel-seq protocol (Hashimshony et al., 2012, 2016) to generate single-cell cDNA libraries using the 3′UTR tail of the transcripts.

In order to establish the efficiency of the approach we used RT primers with unique molecular identifiers and defined amounts of spiked-in ERCC standard RNAs. We could reliably and reproducibly detect ~10–15% of the spiked-in RNA molecules (Figure S1A) with a 50% chance to detect 1 molecule out of 5 spiked-in RNA molecules (Figure S1B). Since the cel-seq method processes barcoded single cell transcriptomes in parallel yielding a combined final sequencing library using IVT and PCR amplification, we tested cross-contamination between barcoded transcriptomes by parallel processing of patch-clamp harvested rat cortical neurons and measuring the contamination of rat mitochondrial DNA sequences in barcoded mouse neuron transcriptomes (see section Methods). The median cross-contamination was 0.0145% between individual barcoded single cell transcriptomes (Figure S1C). Contamination may also arise by accidentally harvesting surrounding tissue besides the cell of interest. Since GABAergic and glutamatergic neurons are heavily intermingled in cortex yet express class specific transcripts, e.g., Gad1, Gad2, Slc32a1 in GABAergic neurons and Slc17a7 in glutamatergic neurons, we estimated contamination by assessing the presence of GABAergic transcripts in glutamatergic neurons and vice versa. GABAergic contamination in glutamatergic neurons and glutamatergic contamination in GABAergic neurons was on average 1.5% ± 0.1(mean ± SEM) and 1.4% ± 0.2(mean ± SEM), respectively, similar to published single cell datasets (see section Methods). Thus, contamination during library preparation and cell harvest are negligibly small and similar across different studies.

To assess technical and biological variability of our single-cell RNA-seq approach we compared technical noise (ERCC spike-ins standards added equally to each sample) and biological variability in SOM/Cbln4 expressing GABAergic neurons harvested in layer 2/3 of mouse visual cortex using the Cre-driver line for SOM GABAergic neurons (Taniguchi et al., 2011) on a gene by gene basis (Brennecke et al., 2013). The layer 2/3 SOM/Cbln4 neurons, a transcriptionally homogeneous neuronal cell-type (Tasic et al., 2016), allow us to estimate variability resulting from our RNA harvesting approach in combination with cell-to-cell biological variability. Technical (ERCC standards) and harvesting/biological (SOM/Cbln4 neurons) variability generally increased with decreasing gene expression (Figure S1D) and was similar to a recently published dataset of the same neuronal cell-type (Figure S1E; Tasic et al., 2016).

The overall technical correlation between samples [ERCC Pearson correlation: 0.997 ± 0.002 mean ± stdev for tpm; 0.896 ± 0.03 for log10(tpm)] and harvesting/biological correlation between individual cells [SOM/Cbln4 Pearson correlation: 0.79 ± 0.126 mean ± stdev for tpm; 0.594 ± 0.022 for log10(tpm)] was similar to published single cell RNAseq data [Tasic et al. ERCC Pearson correlation: 0.985 ± 0.016 mean ± stdev for rpkm; 0.852 ± 0.04 for log10(rpkm); Tasic et al. SOM/Cbln4 Pearson correlation: 0.839 ± 0.115 mean ± stdev for rpkm; 0.588 ± 0.024 for log10(rpkm); Figure S1F].

Thus, single cell RNA-seq from patched cells consistently reports gene expression.

### Validating RNA-seq of patched neurons through distinct gabaergic neurons

**Figure 1:**
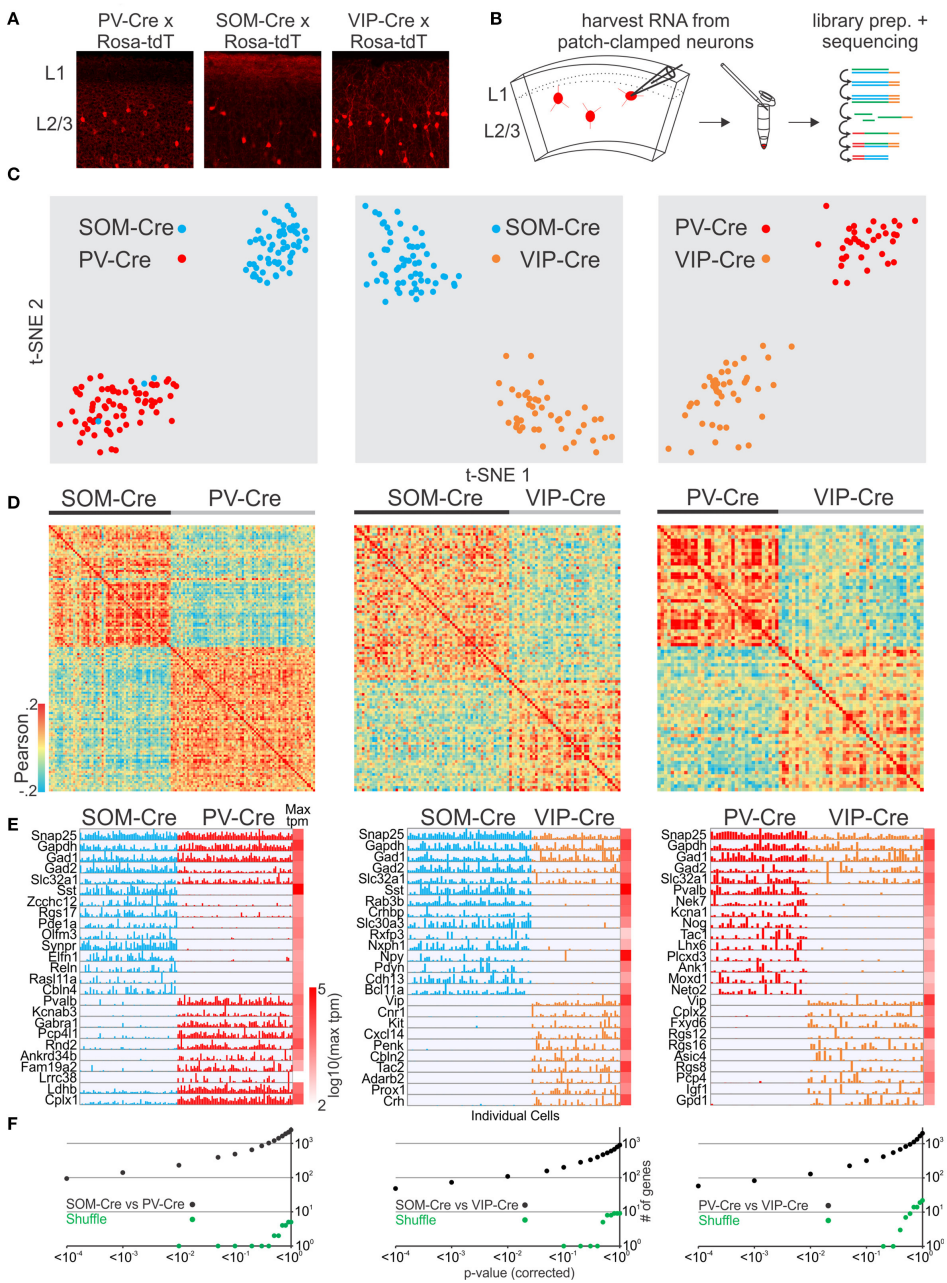
Single cell RNA-seq from patched neurons distinguishes mouse Cre-line defined GABAergic cell-types. **(A)** Fluorescent pictures of PV-Cre, SOM-Cre, and VIP-Cre crossed with a tdTomato reporter showing sparse neuronal labeling in mouse visual cortex layer 2/3. **(B)** Schematic of workflow. Fluorescent neurons from acute slices of mouse cortex were patch-clamped and the interior of the cell sucked into the tip of the patch pipette and subsequently expelled into a small volume tube. CDNA libraries of individual cells were generated and sequenced using high-throughput sequencing. **(C)** Two-dimensional t-SNE representation of gene expression for cell population comparisons (left: PV-Cre vs. SOM-Cre, middle: SOM-Cre vs. VIP-Cre, right: PV-Cre vs. VIP-Cre). Cells are color coded according to their Cre-line correspondence. **(D)** Pairwise comparison of GABAergic cells (left: PV-Cre vs. SOM-Cre, middle: SOM-Cre vs. VIP-Cre, right: PV-Cre vs. VIP-Cre). Shown are color coded heat maps of pairwise Pearson correlation coefficients of the log-normalized z-score expression level (tpm) of the most variable genes (identified after Brennecke et al., 2013). **(E)** Select examples of housekeeping and differentially expressed genes as identified by SCDE (rows) and individual cells (columns) ordered by Cre-line defined genetic cell-type (left: PV-Cre vs. SOM-Cre, middle: SOM-Cre vs. VIP-Cre, right: PV-Cre vs. VIP-Cre). Expression level for each cell is shown (linear scale, max tpm value color coded on the right). **(F)** Plot of number of genes identified as differentially expressed according to their respective *p*-value (SCDE analysis). Black data points represent the original cell distribution (left: PV-Cre vs. SOM-Cre, middle: SOM-Cre vs. VIP-Cre, right: PV-Cre vs. VIP-Cre) and green data points show one representative randomly shuffled distribution. Number of (cells/mice): PV-Cre(71/3), SOM-Cre(60/3), VIP-Cre(43/3).

We next tested whether we could reliably distinguish molecularly established GABAergic interneuron cell classes. We patched neurons to harvest RNA from 3 non-overlapping interneuron cell classes defined by transgenic Cre-driver lines for PV, SOM, and VIP GABAergic interneurons (Hippenmeyer et al., 2005; Taniguchi et al., 2011; Pfeffer et al., 2013) crossed to the Rosa-tdTomato reporter (Figures 1A,B) and identified several thousand genes per neuron (Figure S1G, see also for all other cell comparisons). We then compared the single-cell transcriptomes of PVCre, SOMCre, and VIPCre interneurons. First we identified highly variable genes (Brennecke et al., 2013) which were then used to assess the similarity of gene expression in individual cells using t-distributed stochastic neighbor embedding (t-SNE), principal component analysis (PCA) and Pearson correlation. As expected our analysis shows that t-SNE (Figure 1C), PCA (Figure S2) and Pearson correlation analysis (Figure 1D) separate cells based on variable gene expression into clusters corresponding to the respective molecular GABAergic interneuron class. In order to assess molecular differences between the 3 interneuron classes we computed gene by gene differential expression by comparing the mean and variance of transcript expression across all cells in a pairwise fashion (PVCre vs. SOMCre, PVCre vs. VIPCre, SOMCre vs. VIPCre). Single cell differential expression (SCDE) (Kharchenko et al., 2014) and sigma-scores (see section Methods) were used to identify differentially expressed genes and tested against randomly shuffled cells. SCDE (Figures 1E,F) and sigma-scores (Figure S3) yielded hundreds of differentially expressed genes. These results are consistent with PVCre, SOMCre, and VIPCre cells comprising molecularly distinct GABAergic cell classes confirming previous studies (Pfeffer et al., 2013; Tasic et al., 2016) and validating our single-cell RNA-seq approach.

Finally, we compared the expression patterns of differentially expressed genes from our dataset with the recently published gene expression pattern of GABAergic cell-types in mouse visual cortex (Tasic et al., 2016). We first matched the cells in our dataset to the cell-type scheme of Tasic et al. by classifying them using the previously identified differentially expressed genes and molecular markers provided by Tasic et al. We then selected cells from the Tasic et al. dataset corresponding to our classified cells to assemble matching PV-Tasic, SOM-Tasic, and VIP-Tasic groups. We identified differentially expressed genes using SCDE in a pairwise fashion (PV-Tasic vs. SOM-Tasic; PV-Tasic vs. VIP-Tasic; SOM-Tasic vs. VIP-Tasic). Subsequently, we compared the expression of differentially expressed genes between our and the Tasic et al. dataset side by side (Figures S4–S6, Table S1). The direct comparison shows that many genes identified as differentially expressed (SCDE cutoff probability: *p* < 0.01) overlapped between the datasets. Genes with significant differential expression in only one dataset showed qualitative correspondence with the other, as visualized in the gene expression heat maps (Figures S4–S6). The Tasic et al. dataset identified more genes with significant differential expression between the respective cell groups (Figures S4–S6, Table S1). Overall, the differential gene expression between PVCre, SOMCre, and VIPCre cells harvested through patch-clamp in this study is highly similar to the Tasic et al. dataset. This establishes single cell RNA-seq of patch-clamped neurons as a reliable method to distinguish molecularly distinct cell classes of GABAergic neurons.

### Validating RNA-seq of patched neurons through spatially distinct glutamatergic neurons

**Figure 2:**
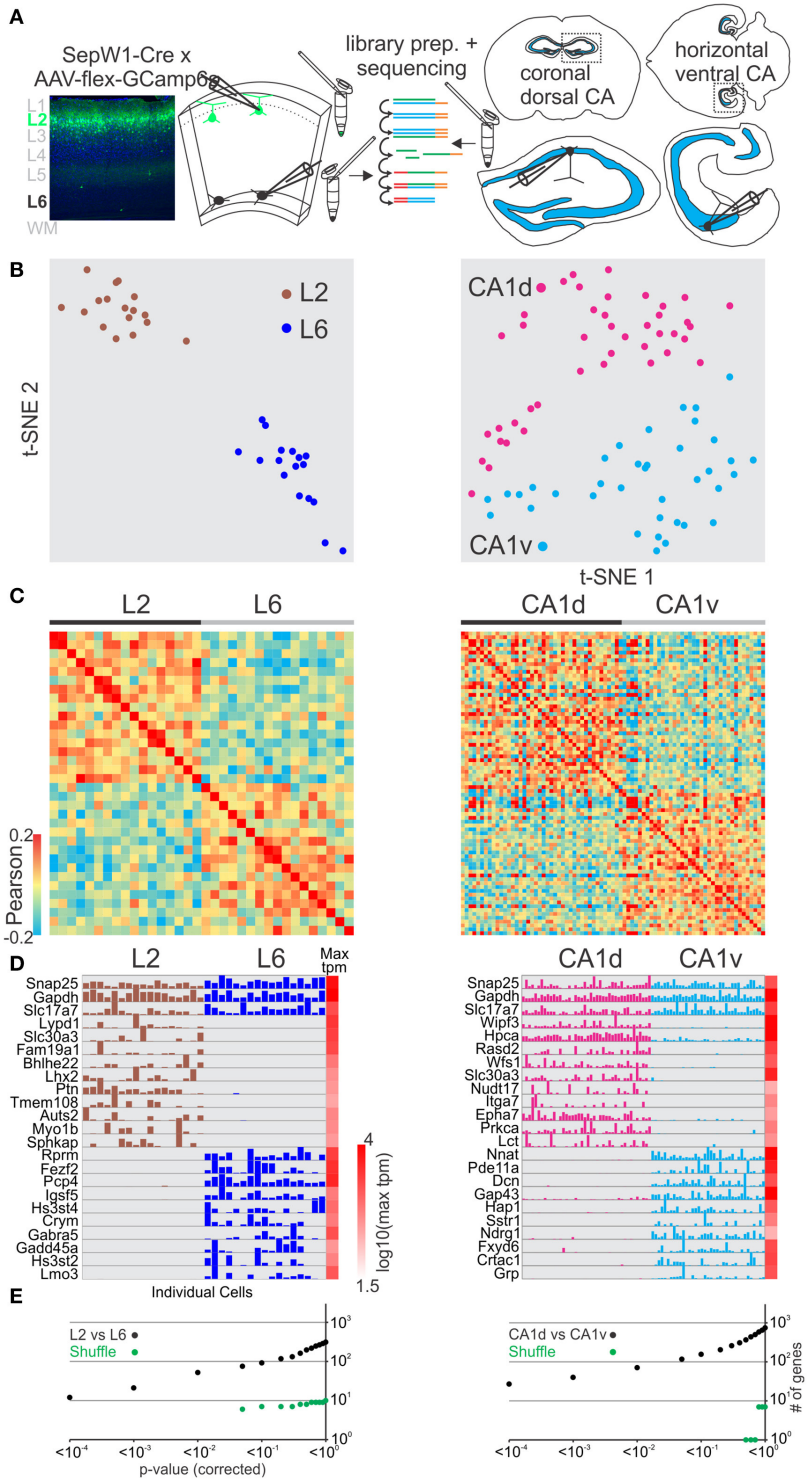
Single cell RNA-seq from patched neurons distinguishes spatially distributed glutamatergic cells. **(A)** Schematic of workflow. Neurons from spatially separate vertical layers of mouse visual cortex (L2, as defined by the SepW1-Cre line; L6, defined as cells of deeper L6 at the border to the white matter) and CA1 pyramidal cells from dorsal (CA1d) and ventral (CA1v) poles of the mouse hippocampus were patch-clamped and the interior of the cell sucked into the tip of the patch pipette and subsequently expelled into a small volume tube. CDNA libraries of individual cells were generated and sequenced using high-throughput sequencing. **(B)** Two-dimensional t-SNE representation of gene expression for cell population comparisons (left: L2 vs. L6, right: CA1d vs. CA1v for **B–E**). Cells are color coded according to their cell population correspondence. **(C)** Pairwise comparison of spatially distinct glutamatergic cells. Shown are color coded heat maps of pairwise Pearson correlation coefficients of the log-normalized z-score expression level (tpm) of the most variable genes (identified after Brennecke et al., 2013). **(D)** Select examples of housekeeping and differentially expressed genes as identified by SCDE (rows) and individual cells (columns) ordered by spatial position. Expression level for each cell is shown (linear scale, max tpm value color coded on the right). **(E)** Plot of number of genes identified as differentially expressed according to their respective *p*-value (SCDE analysis). Black data points represent the original cell distribution and green data points show one representative randomly shuffled distribution. L2 refers to SepW1-Cre positive neurons mostly located in upper layer 2 (Figure S3). Number of (cells/mice): L2-L6(17/2-17/2), CA1d-CA1v(42/4-37/4).

We next asked whether excitatory glutamatergic cells separated in space could be distinguished using this method. Cortex is organized vertically into distinct layers that can be discriminated both histologically and through molecular markers (Figure 2A left). We compared pyramidal cells patched and harvested in superficial layer 2 (using the SepW1-Cre line tagged with GFP; below we refer to SepW1-Cre neurons simply as L2 cells, Figure S7) with deep layer 6 pyramidal cells (L6; harvested at the border to the white matter). Using the same analysis described above (for GABAergic cells) we could molecularly separate pyramidal L6 and pyramidal L2 populations using t-SNE, PCA, and Pearson correlation (Figures 2A–C left, Figure S2). SCDE and sigma-score separated L6 and L2 as assessed by the number of differentially expressed genes (Figures 2D,E left, Figure S3).

The hippocampus is horizontally (longitudinally) separated into molecularly distinct dorsal and ventral poles (Strange et al., 2014; Cembrowski et al., 2016a; Shah et al., 2016). We compared pyramidal cells patched and harvested in dorsal CA1 (CA1d) with ventral CA1 (CA1v) (Figure 2A right). Our single cell transcriptome analysis shows that CA1d and CA1v can be readily separated into distinct molecular populations confirming previous results (Figures 2B–E right, Figures S2, S3).

We then compared differentially expressed genes from our dataset with the Tasic et al. dataset for L6-L2 as well as the Cembrowski et al. gene enrichment dataset for CA1d-CA1v (Cembrowski et al., 2016a,b; Figures S8, S9). Similar to the GABAergic neuron analysis above, the Tasic et al. dataset identified more genes with significant differential expression between the respective cell groups (Table S1). The overall similarity of differentially expressed genes was high for the L6-L2 and CA1d-CA1v comparisons. Finally, the layer- or region-specific expression pattern of selected genes revealed with our approach matched the layer- or region-specific expression pattern reported in the Allen *in situ* hybridization mouse brain database (Figures S10A,C). Together, these data demonstrate that single-cell RNA-seq from patched cells allows us to molecularly distinguish glutamatergic neuron populations which differ in their spatial distribution within confined cortical areas.

Our approach establishes single cell RNA-seq of patch-clamped neurons as a simple method to correlate cellular characteristics like spatial location with gene expression. While spatial separations across the vertically layered cortex or the distant ventral and dorsal hippocampal poles are easily resolved, separations within specific cortical areas or across adjacent cortical areas like primary and secondary sensory cortices require additional distinction measures. We used our methodology together with fluorescent tracers to explore and compare the transcriptome of L2 pyramidal neurons in primary and secondary visual cortex.

### RNA-seq of topographically distinct glutamatergic L2 neurons in mouse visual cortex

**Figure 3:**
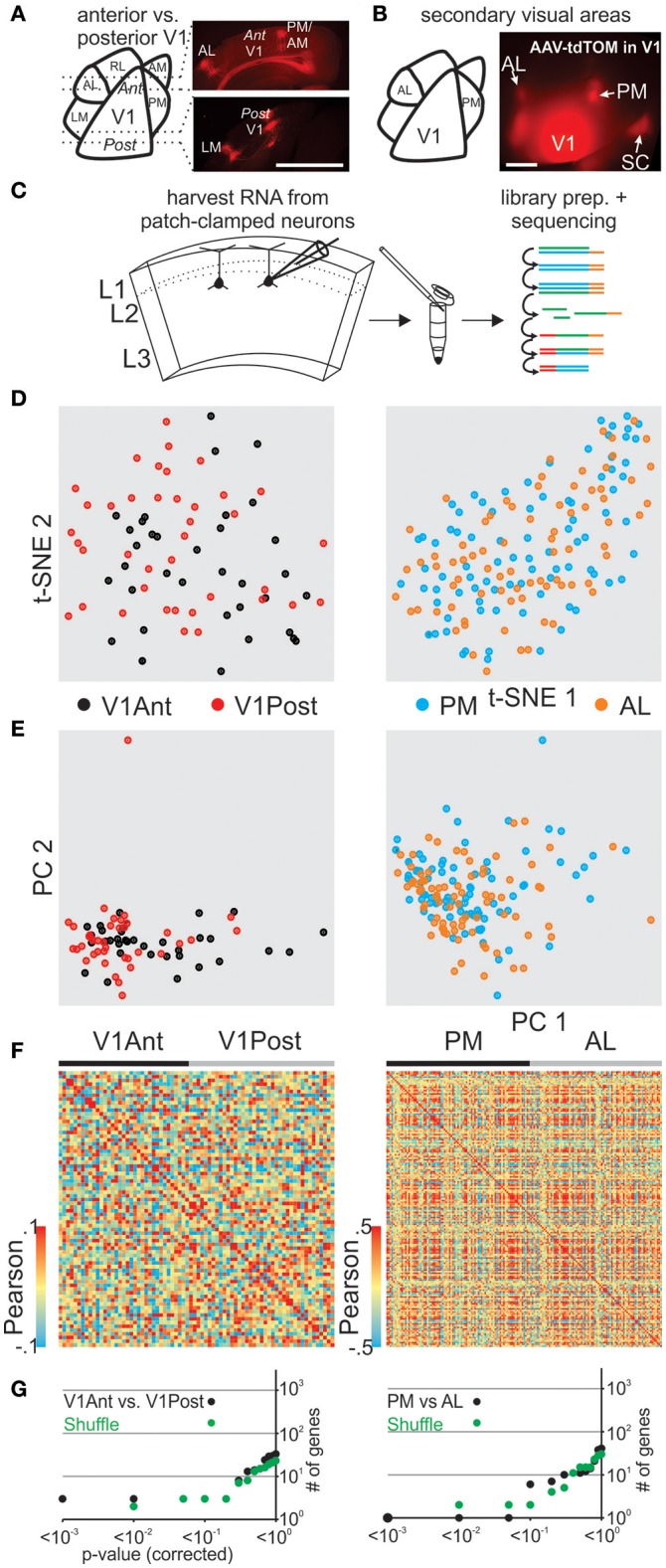
Correlating spatial position of L2 pyramidal neurons with their gene expression pattern in mouse visual cortex. **(A)** Schematic representation of mouse visual cortex (left). Fluorescent viral axonal tracer (tdTomato) was injected in posterior primary visual cortex (V1) and axonal projections to secondary areas can be readily identified in coronal sections delineating posterior and anterior V1 (right, see also in **B** and Figure S7). **(B)** Schematic of mouse visual cortex (left). Fluorescent viral axonal tracer (tdTomato) was injected in posterior primary V1 and secondary areas can be readily identified based on axonal projections (top view on mouse visual cortex, right). **(C)** Schematic of workflow. L2 neurons from spatially separate positions (anterior vs. posterior V1, AL vs. PM) of mouse visual cortex were patch-clamped and the interior of the cell sucked into the tip of the patch pipette and subsequently expelled into a small volume tube. CDNA libraries of individual cells were generated and sequenced using high-throughput sequencing. **(D)** Two-dimensional t-SNE representation of gene expression for cell population comparisons (left: V1Ant vs. V1Post, right: AL vs. PM for **D-G**). Cells are color coded according to their cell population correspondence. **(E)** Two-dimensional PCA representation of gene expression for cell population comparisons. Cells are color coded according to their cell population correspondence. **(F)** Pairwise comparison of spatially distinct L2 glutamatergic cells. Shown are color coded heat maps of pairwise Pearson correlation coefficients of the log-normalized z-score expression level (tpm) of the most variable genes (identified after Brennecke et al., 2013). **(G)** Plot of number of genes identified as differentially expressed according to their respective *p*-value (SCDE analysis). Black data points represent the original cell distribution and green data points show one representative randomly shuffled distribution. L2 refers to glutamatergic neurons in upper layer 2 right below L1. Number of (cells/mice): V1Ant-V1Post(35/3-39/3), PM-AL(85/5-78/5).

Primary visual cortex (V1) receives sensory information in a topographic fashion with the anterior and posterior V1 tuned to inputs from the lower nasal and upper temporal part of the retina, respectively. To determine and compare the transcriptome of pyramidal L2 neurons in the posterior and the anterior portion of V1 we must first delineate the boundaries of V1. Because V1 neurons project to secondary areas that surround V1 (Wang and Burkhalter, 2007; Glickfeld et al., 2013) we used their axonal arborizations to delineate V1 *in vivo*. Upon viral expression of tdTomato in the posterior part of V1, secondary visual areas could be readily identified and coronal slices containing posterior or anterior V1 were used to patch and harvest pyramidal neurons in L2 (Figures 3A,C, Figure S11). Our transcriptomic analysis of patched cells from opposite fields of the visual space did not show clear molecular differences (using t-SNE, PCA, Pearson) between anterior and posterior pyramidal V1 L2 neurons (Figures 3D–F left) and SCDE or sigma-scores could not clearly identify differentially expressed genes when tested against shuffled cell populations (Figure 3G top, Figure S3).

We next determined and compared the transcriptome of pyramidal L2 neurons belonging to the secondary visual areas AL (anterior lateral) and PM (posterior medial; Wang and Burkhalter, 2007; Garrett et al., 2014), two areas that have been shown to differ in their response characteristics to visual stimuli (Andermann et al., 2011; Marshel et al., 2011). As above, we used the axonal arborization of V1 neurons to identify these two secondary visual areas (Wang and Burkhalter, 2007; Glickfeld et al., 2013; Figure 3B, Figure S11). Using coronal slices we patched and harvested L2 pyramidal neurons belonging to either AL or PM and compared their single cell transcriptomes (Figures 3D–F right, Figure 3G bottom, Figure S3). Our transcriptome analysis of AL and PM L2 pyramidal neurons representing distinct secondary visual areas did not yield molecularly distinguishable cell populations.

Together, *in vitro* patch clamp and *in vivo* local fluorescent tracers can be combined to study the relationship between single cell gene expression and spatial location within anatomically precisely delineated cortical structures. Our results suggest that pyramidal L2 neurons belonging to separate secondary visual areas or opposite poles of the primary visual cortex are molecularly similar.

### Validating RNA-seq of patched neurons through distinct l6 projection neurons

**Figure 4:**
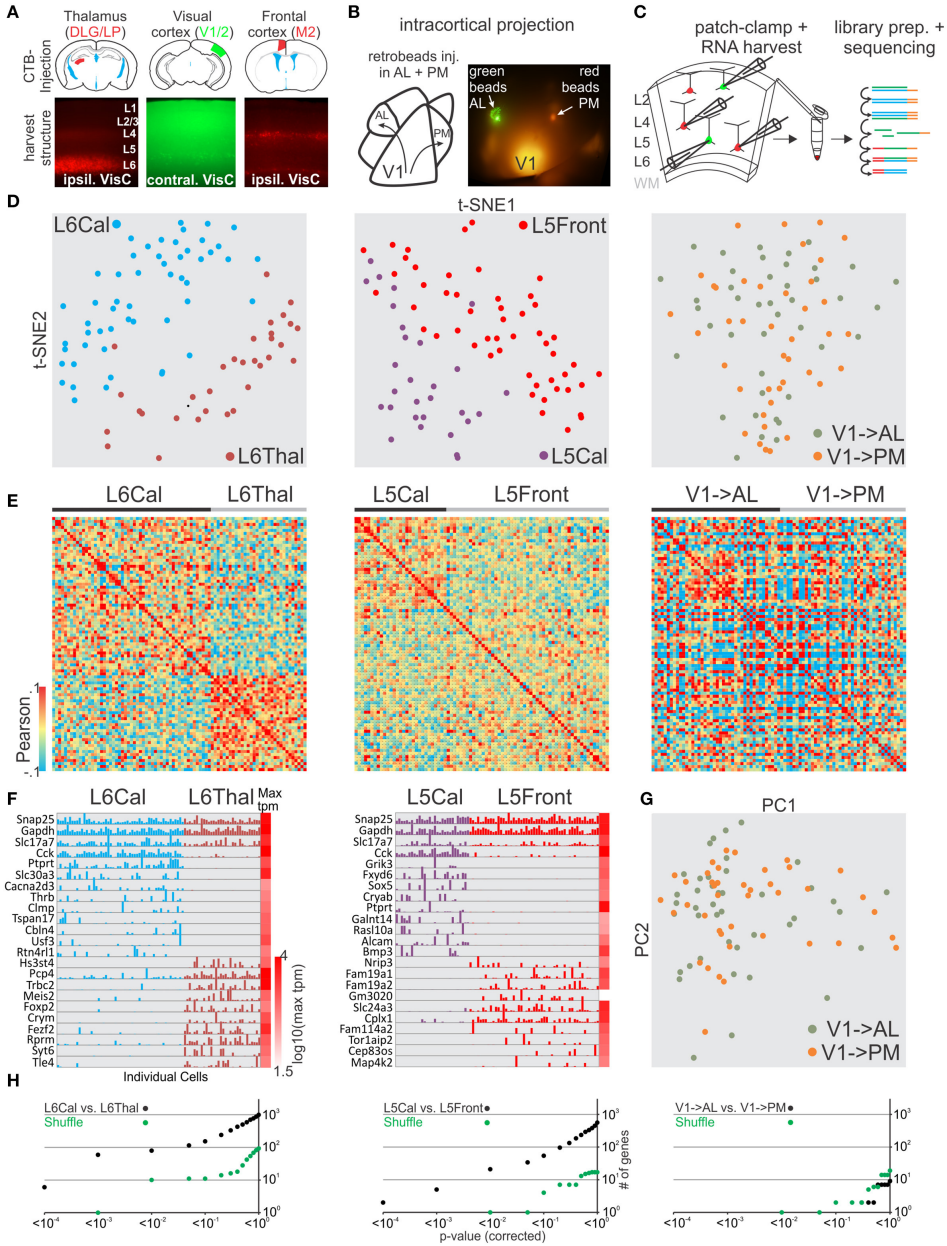
Single cell RNA-seq from patched neurons distinguishes glutamatergic cells defined by their axonal projection. **(A)** Schematic of injection sites (top). Mouse brains were injected with retrograde tracer in known axonal targets of mouse visual cortex projection neurons (frontal cortex, contralateral visual cortex, and thalamus). **(B)** Schematic of mouse visual cortex with axonal projection from posterior V1 to AL and PM. Secondary areas AL and PM were visualized by tdTomato containing axonal projections. Fluorescent latex retro-beads were injected into secondary areas AL and PM. **(C)** Schematic of workflow. Neurons from visual cortex were identified by their accumulation of fluorescent tracer, patch-clamped and the interior of the cell sucked into the tip of the patch pipette and subsequently expelled into a small volume tube. CDNA libraries of individual cells where generated and sequenced using high-throughput sequencing. Neurons were harvested from layer 5 and 6 with callosal (L5Cal, L6Cal), frontal (L5Front), and thalamic (L6Thal) projections and layer 2 with projections to AL (V1->AL) and PM (V1-PM). **(D)** Two-dimensional t-SNE representation of gene expression for cell population comparisons (left: L6Cal vs. L6Thal, middle: L5Cal vs. L5Front, right: V1->AL vs. V1->PM for **D,E,H**). Cells are color coded according to their cell population correspondence. **(E)** Pairwise comparison of distinct glutamatergic projection neurons in visual cortex. Shown are color coded heat maps of pairwise Pearson correlation coefficients of the log-normalized z-score expression level (tpm) of the most variable genes (identified after Brennecke et al., 2013). **(F)** Select examples of housekeeping and differentially expressed genes as identified by SCDE (rows) and individual cells (columns) ordered by projection population. Expression level for each cell is shown (linear scale, max tpm value color coded on the right). **(G)** Two-dimensional PCA representation of gene expression for cell population comparison (V1->AL vs. V1->PM). Cells are color coded according to their cell population correspondence. **(H)** Plot of number of genes identified as differentially expressed according to their respective *p*-value (SCDE analysis). Black data points represent the original cell distribution and green data points show one representative randomly shuffled distribution. Number of (cells/mice): L6Cal-L6Thal(53/4-32/3), L5Cal-L5Front(29/3-51/4), V1->AL-V1->PM(42/5-41/5).

After validating our general approach we tested whether our methodology could distinguish glutamatergic projection neurons belonging to the same layer in primary visual cortex but projecting their axons to cortical or sub-cortical targets. We compared neurons patched and harvested in layer 6 (L6) that send a callosal projection to the contralateral cortex (callosal L6, L6Cal) with L6 neurons that project to the thalamus (L6Thal). L6Cal and L6Thal neurons were retrogradely labeled with fluorescent tracer (CTB conjugated to Alexa 488 or 594) injection into contralateral visual cortex or ipsilateral thalamus, respectively (Figure 4A). In line with previous research (Bortone et al., 2014; Velez-Fort et al., 2014; Tasic et al., 2016) single cell transcriptomic analysis of patched cells showed that L6Cal neurons are molecularly distinct from L6Thal neurons (Figures 4D–F,H left, Figures S2, S3). We compared differentially expressed genes between our dataset and the dataset from Tasic et al. for L6Thal-L6Cal (Figure S12, Table S1). While the Tasic et al. dataset identified more genes with significant differential expression between the respective cell groups the overall similarity of differentially expressed genes was high between the two datasets. Finally, the sublayer-specific expression pattern of selected genes in our two L6 populations matched the layer-specific expression pattern reported in the Allen *in-situ* hybridization mouse brain database (Figure S10B). Our analysis shows that single cell RNA-seq from patched neurons allows us to distinguish among specific projection neuron classes within the same cortical layer.

### RNA-seq of patched L5 and L2 cortico-cortical projection neurons in visual cortex

Our approach establishes single cell RNA-seq of patch-clamped neurons as a simple method to correlate cellular characteristics like spatial location or axonal projection with gene expression. We tested whether this approach would allow us to reveal molecular differences between pyramidal cells located in the same layer within V1 but projecting to distinct cortical targets, i.e., whether we could reveal differences between cortico-cortical pyramidal cells (rather than between cortico-cortical and cortico-subcortical, as above). We focused on L5 and L2 pyramidal cells. L5 cortico-cortical cells in V1 can be distinguished based on whether they project to the contralateral hemisphere through the corpus callosum (L5Cal) or ipsilateral to the frontal cortex (L5Front). L2 pyramidal cells in V1 can be distinguished based on the secondary visual area they project to, for example AL (V1->AL) and PM (V1->PM; Glickfeld et al., 2013). To label L5Cal and L5Front we injected retrograde fluorescent tracers (CTB conjugated to alexa 488 or 594) into contralateral visual cortex or ipsilateral frontal cortex, respectively (see section Methods). L5Cal neurons were found throughout L5 while L5Front neurons were more concentrated at the intersection between L5 and L4 (Figure 4A). Transcriptomic analysis showed that L5Cal neurons are molecularly distinct from L5Front neurons (Figures 4D–F,H middle, Figures S2, S3). We compared differentially expressed genes between our dataset and the dataset of Tasic et al. for L5Front-L5Cal (Figure S13, Table S1) and found overall similarity. However, the similarity was weaker than other comparisons (e.g., L6Cal-L6Thal) particularly for genes that identified as differentially expressed in our dataset using SCDE. Similar to L6Cal neurons and contrary to L6Thal and L5Front neurons, L5Cal neurons expressed high levels of cholecystokinin (Cck) suggesting that callosal L5/6 neurons can be identified using Cck as a marker gene. Finally, the sublayer-specific expression pattern of selected genes in our two L5 populations matched the layer-specific expression pattern reported in the Allen *in-situ* hybridization mouse brain database (Figure S10B). Our results show that visual cortex L5 neurons extending their axons to ipsilateral frontal cortex or contralateral visual cortex are molecularly distinct.

To label V1->AL and V1->PM we first identified the secondary visual areas AL and PM using the axonal arborizations of V1 neurons, as described above. We subsequently injected green or red fluorescent retrograde tracer beads into the secondary areas AL and PM, respectively, to retrogradely label the projection neurons in V1 *in vivo* (Figure 4B). We patched and harvested RNA from retrogradely labeled L2 V1->AL and V1->PM pyramidal cells in slices of V1. Retrograde labeling in neurons was sparse and none of the neurons showed both red and green bead labeling, consistent with the selective targeting of secondary visual areas by V1 neurons described previously (Glickfeld et al., 2013). In contrast to the transcriptional differences observed between L5 cells projecting to contralateral and frontal cortex, L2 pyramidal neurons projecting to AL or PM showed no clear separation into molecularly distinguishable cell populations (Figures 4C–H right, Figure 4G, Figure S3).

Thus, *in vitro* patch clamp and *in vivo* fluorescent tracers can be combined to study the relationship between gene expression of individual neurons and axonal projection with the spatial resolution provided by visually guided patch pipette targeting. Our results, including L6 thalamic and callosal projection neurons, indicate that target specificity is a strong predictor of molecular identity of L5 and L6 long-range projection neurons. In contrast, L2 pyramidal neurons projecting to separate secondary visual areas have a much more similar gene expression pattern.

### Combining functional identification of single neurons *in vivo* with transcriptional analysis

**Figure 5:**
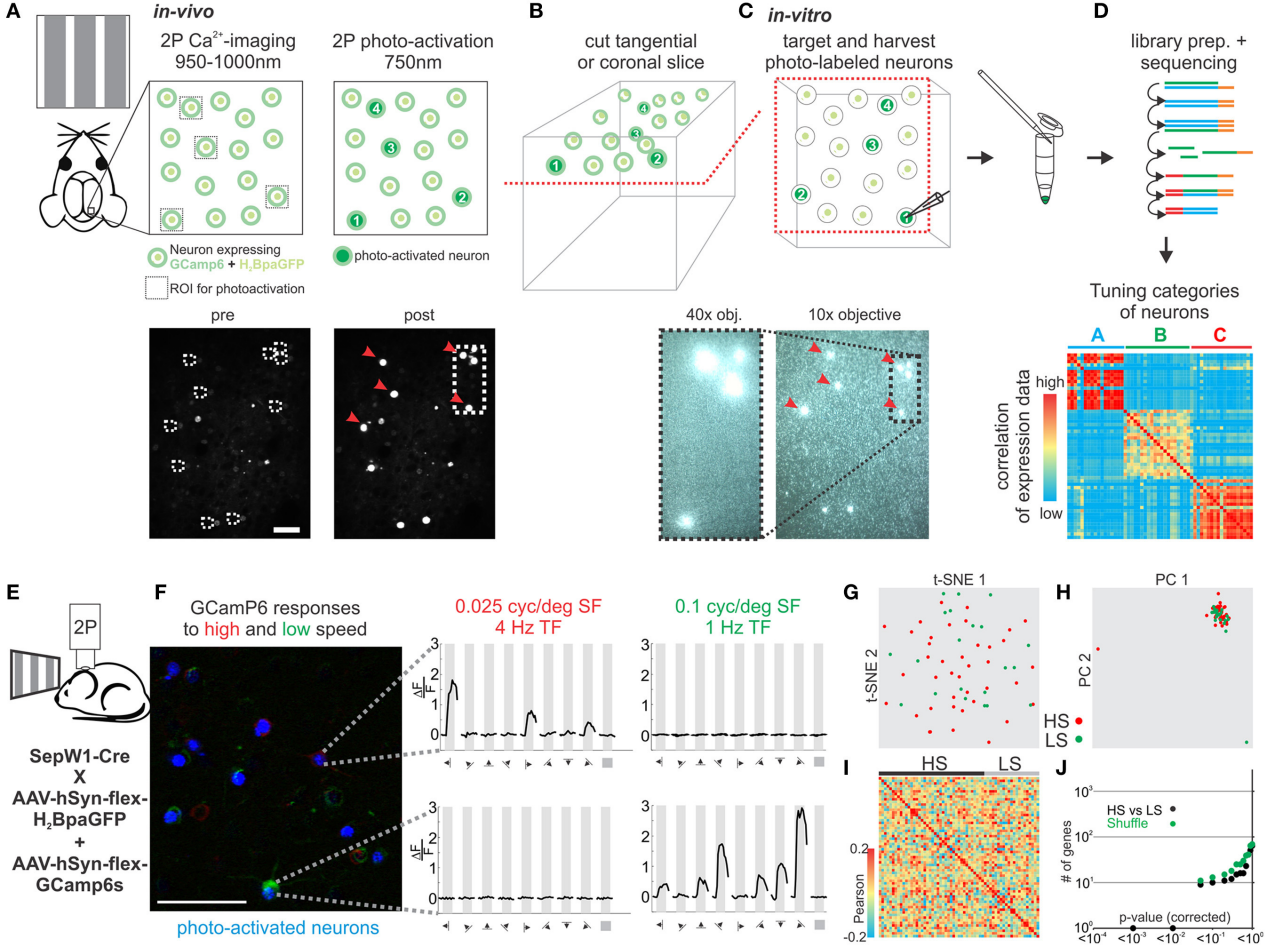
Correlating *in vivo* tuning properties of individual neurons with their gene expression pattern. **(A–D)** Experimental setup and workflow. **(A)** Mice expressing GCamP6 and photo-activatable nucleus bound GFP in neurons of the brain are imaged *in vivo* during visual stimulation. Two-photon imaging is performed at 950–1,000 nm. After imaging neurons are photo-tagged by scanning select neurons briefly at 750–800 nm photoactivating the GFP. **(B)** After *in vivo* imaging acute slices of the imaged area are cut in coronal or tangential orientation. Slices are placed under a slice physiology microscope and photo-tagged neurons can be easily visualized and matched to the *in vivo* registration. Identified photo-tagged neurons are then patched and the RNA is extracted into the patch-pipette. **(C)** The harvested RNA is expelled into a tube and a sequencing library constructed which is finally deep sequenced. **(D)** The transcriptomes of functionally characterized neurons are correlated to identify molecular cell-types with specific *in vivo* functional tuning properties. **(E)** Schematic of *in vivo* imaging. **(F)** Color coded 2P imaging of neurons in primary visual cortex responding to high speed visual stimuli (red) or low speed visual stimuli (green) and their subsequent photo-tagging (blue). Right—plots of example neurons responding to either high (red) or low (green) speed visual stimuli. Plots show calcium responses at specific grating directions. Scalebar: 50 μm. **(G)** Two-dimensional t-SNE representation of gene expression for cell population comparisons. Cells are color coded according to their cell population correspondence (red: HS-high speed, green: LS-low speed). **(H)** Two-dimensional PCA representation of gene expression for cell population comparisons. Cells are color coded according to their cell population correspondence (red: HS-high speed, green: LS-low speed). **(I)** Pairwise comparison of distinct glutamatergic projection neurons in visual cortex. Shown are color coded heat maps of pairwise Pearson correlation coefficients of the log-normalized z-score expression level (tpm) of the most variable genes (identified after Brennecke et al., 2013). **(J)** Plot of number of genes identified as differentially expressed according to their respective *p*-value (SCDE analysis). Black data points represent the original cell distribution and green data points show one representative randomly shuffled distribution. Number of (cells/mice): HS(40/10), LS(21/10).

Neurons in sensory cortex can be classified based on how they respond to sensory stimulation, i.e., based on their sensory tuning properties. To date, no simple and generally applicable method exists to determine the gene expression pattern of a neuron whose response properties to a sensory stimulus are known. We modified and extended an approach previously developed in our lab (Lien and Scanziani, 2011) by assessing the tuning properties of individual neurons *in vivo* through calcium imaging and subsequently establishing their transcriptional profile by harvesting their RNA *in vitro* (Figures 5A–D). We applied our approach to evaluate transcriptional differences in neurons tuned to either slow or fast visual moving gratings in mouse visual cortex. Using laser scanning 2-photon microscopy (950–1,000 nm) we imaged calcium transients in L2 neurons in V1 of anesthetized mice conditionally expressing the calcium indicator GCamP6s and the histone-bound photo-activatable GFP (H_2_BpaGFP) in glutamatergic Sepw1-Cre mice (Figure 5E). L2 neurons responded robustly to visual stimuli presented on a computer monitor. We functionally separated neurons based on their selective responses to slow (0.1 cyc/deg spatial frequency, 1 Hz temporal frequency) and fast (0.025 cyc/deg spatial frequency, 4 Hz temporal frequency) moving gratings presented in 8 directions (0–315°; Figure 5F). The few neurons that responded significantly to both stimuli were excluded. Neurons of interest were subsequently scanned at 750–800 nm in order to photo-activate the H_2_BpaGFP. We cut coronal slices from the imaged region of V1 and, using a regular slice fluorescence microscope, we registered the photo-labeled neurons in the slice with those imaged *in vivo*. Thus, neurons with known response properties to visual simulation could be patched *in vitro* and their RNA harvested. Our analysis of single cell transcriptomes of V1 L2 pyramidal neurons tuned to high speed or low speed moving gratings showed no clear separation into molecularly distinguishable cell populations (Figures 5G–J, Figure S3).

Our methodology allows us for the first time to study tuning properties of individual neurons *in vivo* and subsequently assess their transcriptomes by patching those same neurons *in vitro* to harvest their RNA. When applied to study the relationship of speed tuning and molecular identity in mouse V1 our results suggest that pyramidal L2 neurons tuned to fast or slow stimuli are molecularly very similar.

## Discussion

Being able to correlate functional, positional, and projectional properties of individual neurons with their gene expression pattern is a critical step toward a complete understanding of the functional organization of the brain. Recently, several publications have used dissociated cells of bulk brain tissue with subsequent single cell transcriptomic profiling to molecularly classify cells into distinct cell types (Macosko et al., 2015; Zeisel et al., 2015; Tasic et al., 2016). This powerful approach, while highly scalable, lacks however information about several cell properties that are classically used to define cell types. The complex fine-scale circuitry of the nervous systems requires the ability to perform single cell transcriptional profiling of neurons whose precise positional, anatomical, and functional integration in the circuit are known. Here we have developed approaches that allow us to identify and label cortical neurons with specific positions, projections, and response properties to sensory stimulation *in vivo*, and harvest their RNA through patch clamp of single neurons *in vitro* for transcriptional profiling. We demonstrate the applicability and validity of our approaches using various cell classes of mouse visual cortex and the hippocampus that differ in their genetic labeling, their spatial position, or their long-range axonal projections. We explore the gene expression profile in visual cortical layer 2 and layer 5 pyramidal neurons that can be distinguished based on their position, cortical axonal targets, or response to visual stimulation.

We extensively validate our approach by demonstrating our ability to molecularly separate multiple previously established molecular cell-types. We extend our validation by directly comparing our results with a previous single cell study on visual cortical neurons (Tasic et al., 2016) and a previous hippocampus CA1 gene enrichment study (Cembrowski et al., 2016a). We find that our results identifying differentially expressed genes reproduce the previous findings with high similarity. Discrepancies in differential gene expression between our and the published datasets might be due to differences in mouse genetic backgrounds, mouse age at sampling, sampling procedures (patch clamp harvest from acute slices vs. bulk dissected single cells), or library preparation. The higher number of identified differentially expressed genes from the Tasic et al. and Cembrowski et al. studies is likely a result of deeper sequencing and a more consistent RNA capture from isolated single cells or pooled cells (see also section Methods). The comparison of L5 frontal vs. callosal projection types with Tasic et al. molecular cell-types showed only an approximate correspondence of differentially expressed gene patterns. This might be due to a more complex underlying molecular cell-type combination for each projection type or L5 frontal and callosal projecting neurons belonging to not yet defined molecular subtypes. Nevertheless, the high degree of similarity between our and other datasets validates our approach using patch-clamp and RNAseq in the study of molecular cell-types and their matching onto neuronal parameters. In future work, systematically comparing molecular profiles of neurons whose anatomy, connectivity, and function have been determined in parallel will be required for a more comprehensive understanding of molecular cell-types within their respective neural circuits.

We have successfully combined single cell patch clamp transcriptional profiling with functional imaging *in vivo*. This allowed us to reveal the gene expression pattern of individual neurons identified by their specific response properties to sensory stimulation. For this we have extended a previous approach (Lien and Scanziani, 2011) that combines the single cell resolution of two photon laser scanning microscopy for functional analysis *in vivo*, with the single cell resolution of *in vitro* visually guided patch-clamp for RNA harvesting. This method allows one to correlate, in principle, any functional attribute of a neuron that is optically addressable *in vivo* with its gene expression pattern. This approach could be extended to longitudinal *in vivo* imaging studies (Huber et al., 2012; Poort et al., 2015) to address time or condition dependent changes in transcriptional profiles in relation to behavior, learning, and memory.

The ability to correlate the transcriptional profile of individual neurons with other properties, like their positional, anatomical, or functional characteristics may help separate molecularly distinct neuronal classes that would not emerge with unsupervised clustering algorithms based purely on gene expression profiles. For example, unsupervised clustering can miss distinctions that follow gradient expression of genes like the dorsal-ventral axis of hippocampal CA1 neurons (Zeisel et al., 2015; Cembrowski et al., 2016a). Similarly, expression of cholecystokinin (Cck) can be found across multiple molecular cell types but highly correlates with callosal projecting neurons in L5 and L6 (Figure 4).

Our validation and exploration experiments allow us to separate GABAergic and glutamatergic neurons into transcriptionally distinct categories (Figures 1, 2, 4) and correlate those categories with anatomical information like position or axonal projection. We find that the distinct functional and anatomical characteristics of superficial L2 pyramidal neurons of primary and secondary visual cortex do not correlate well with particular gene expression profiles. Consistent with this result, recent work using transcriptional profiling of cortical neurons failed to identify multiple glutamatergic molecular cell types within L2 and L3 (Zeisel et al., 2015; Tasic et al., 2016). It is possible that further exploration of the functional and anatomical parameter space of L2 pyramidal neurons, including developmentally earlier time points when synaptic connections are first established (Chou et al., 2013), might unravel a predictive transcriptional logic. Furthermore, we cannot exclude that the RNA sequencing used here missed existing correlations: Deeper sequencing, larger samples or the inclusion of splice variants, micro- or non-coding RNAs, or genome modifications (e.g., methylome) may allow one to reveal more subtle correlations [e.g., molecular spatial gradients (Cembrowski et al., 2016a) or small differences in global gene expression (Hawrylycz et al., 2010)] that were not captured in this study.

Research has shown that molecularly defined cortical neuron-types strongly correlate with specific *in-vivo* response properties. For example, in mouse visual cortex PV interneurons are tuned to smaller visual fields than SOM interneurons and the fraction of neurons tuned to oblique or cardinal orientations is unbiased in layer 4 while layer 6 pyramidal neurons favor cardinal orientations (Adesnik et al., 2012; Andermann et al., 2013). While it is not understood how genetic programs define the corresponding underlying synaptic circuits, it is unlikely that all tuning properties of every neuron are genetically predetermined. The approaches described here will help dissect genes and synaptic circuits that define neuronal characteristics.

Recently, patch clamp with single cell RNAseq was introduced to correlate intrinsic spiking characteristics and gene expression in individual neurons (Cadwell et al., 2016; Fuzik et al., 2016). Here we develop and extend this approach to reveal the transcriptome of individual neurons whose anatomical characteristics and response properties to sensory stimulation have been predetermined *in vivo*. Furthermore, we rigorously validate our approach using several hundred neurons from multiple GABAergic and glutamatergic cell classes. Our approach allows us to obtain a coherent and multidimensional definition of individual neurons and will help teasing apart the fundamental functional, anatomical, and genetic organizational principles of neural circuits.

## Methods

### Animals

Mice (postnatal day p30 and older) of mixed backgrounds (129S2/SvPasCrl, c57bl6, CD-1) and sexes were group-housed in the vivarium under reversed light/dark (12/12 h) conditions. One Sprague-Dawley rats (postnatal day p28 and older) group-housed in the vivarium under reversed light/dark (12/12 h) conditions were used to obtain slices for contamination experiments. Animals used had no previous history of drug administration, surgery, or behavioral testing. All procedures were conducted in accordance with the National Institutes of Health guidelines and with the approval of the Committee on Animal Care at the University of California, San Diego. Transgenic mice used were: ROSA-tdTomato (Madisen et al., 2010), Pvalb-Cre (Hippenmeyer et al., 2005), Sst-Cre (Taniguchi et al., 2011), VIP-Cre (Taniguchi et al., 2011), SepW1-Cre (Gensat: NP39-Cre).

### Virus injections in pups

Adeno-associated viruses (AAVs) for GCAMP6s were acquired from the University of Pennsylvania Viral Vector Core: AAV2/9.hSyn.flex.GCAMP6s.SV40 and for H_2_BpaGFP was acquired from the Salk vector core: AAV2/9.hSyn.flex.H2BpaGFP.SV40. Virus mixtures (GCAMP6s/H2BpaGFP) were loaded in a beveled sharp micropipette mounted on a Nanoject II (Drumond) attached to a micromanipulator. GCAMP6s/H_2_BpaGFP viruses were injected into newborn pups (p0-p2) of SepW1-Cre mice. Newborn mice were anesthetized on ice and secured into a molded platform. Three 23 nl boli of virus were injected unilaterally at each of three medial–lateral locations in V1 and one depth (300 μm).

### Adult viral and retrograde tracer injections

To target specific secondary visual cortices with retrograde tracers, mice >p35 underwent two subsequent surgeries.

During the first surgery, a volume of 50 nl of adeno-associated virus AAV1.CAG.TdTomato.WPRE.SV40 (titer 1.19^*^10^13^, Penn Vector Core) was injected at a rate of 20 nl^*^min^−1^ into the left primary visual cortex (2,700 μm from midline, over the lambdoid suture, 500 μm depth), as previously described (Olsen et al., 2012).

The second surgery was performed 15 days after the viral injection. The skull covering primary and secondary visual cortices was thinned using a drill and moistened with sterile saline solution. Through transcranial epifluorescence, the position of secondary visual areas was determined by the identification of the axonal projections of the Td-tomato expressing V1 neurons (Wang and Burkhalter, 2007). Small craniotomies (300 μm diameter) were performed over the identified higher visual cortices, where a bolus (40 nl) of fluorescent retrograde tracers (Green Retrobeads™ IX or Red Retrobeads™ IX, Lumafluor) was injected at 300 μm depth through a beveled micropipette, at 20 nl^*^min^−1^ rate. The skin was then sutured and the animal treated with buprenorphine (0.1 mg kg^−1^, subcutaneous injection).

Cholera toxin B conjugated to alexa 488 or 594 (CTB-488, CTB-594, Life Technologies) were dissolved in standard PBS (0.5%). Craniotomies to target thalamus, contralateral visual cortex, and frontal cortex were performed as described above with the following locations (thalamus: 2 mm posterior from bregma, 2 mm lateral from midline, depth: 2.9–3 mm; contralateral visual cortex: 3 mm lateral from midline, 400 μm anterior from lambda, depth: 400 μm; ipsilateral frontal cortex: over the bregma suture, 100 μm from midline, depth: 250 μm). A bolus of 20 nl was injected through a beveled micropipette.

### Slice preparation

Mice aged P35 or older were anesthetized with ketamine and xylazine (100 and 10 mg^*^kg^−1^, respectively), perfused transcardially with cold sucrose solution (in mM: NaCl, 83; KCl, 2.5; MgSO4, 3.3; NaH2PO4, 1; NaHCO3, 26.2; D-glucose, 22; sucrose, 72; and CaCl2, 0.5, bubbled with 95% O2 and 5% CO2) and decapitated, and the visual cortex was cut into 300 μm coronal or tangential sections in cold sucrose solution. Slices were incubated in sucrose solution in a submerged chamber at 34°C for 30 min and then at room temperature (21°C) until used for RNA harvest from patch-clamped cells. For cross-contamination experiments cortical slices were prepared from rats (Sprague-Dawley) 4 weeks and older.

### Single cell RNA harvest

RNA was harvested as described previously (Pfeffer et al., 2013). In brief, slices were mounted under a 40x 0.8 NA water lens and superfused with sucrose solution (same as in slice preparation). Cells were identified using GFP or tdTomato fluorescence and visualized with IR-DIC. Cells were then patched with regular RNAse free patch pipettes (2–4 MOhm resistance) in whole-cell mode using the voltage-clamp configuration. After seal establishment and break-in the cell was held at −70 mV and the cell content including cell nucleus was harvested using negative pressure and expelled in an Eppendorf tube and frozen at −7°C. Intracellular solution was RNAse free: in mM: CsMeSO4, 115; NaCl, 4; HEPES, 10; Na3GTP, 0.3; MgATP, 4; EGTA, 0.3; BAPTA(4Cs), 10; adjusted to pH 7.4 with CsOH; mOsm 295. The procedures lasted ~2–3 min per cell (from targeting to tube freezing).

### Single cell RNA-seq

Single cell RNA-seq was carried out following the Cel-seq protocol (Hashimshony et al., 2012, 2016). RT and second strand synthesis were performed using Superscript III (Invitrogen) and second strand synthesis kit (Invitrogen), respectively, before IVT amplification (Maxiscript T7, Ambion). Ninety-six samples (up to 94 cells and 2–8 control samples) were handled in parallel to construct one sequencing library. After library fragmentation (Mg-Acetat + heat) and 3′ adaptor attachment, 11–15 cycles of PCR were used for library amplification (final concentration >1 ng/microL, library peak fragment size ~350 nt). Libraries were sequenced using Illumina HiSeq 2500 and HiSeq 4000 at the UCSD genomics core (1st read 13 cycles for the barcode, 2nd read 50 cycles for the 3′ end of the transcript).

Reads were demultiplexed, trimmed, and filtered (prinseq, parameters: –lc_method dust –lc_threshold 15 –trim_left 7) before mapping (bowtie2, standard parameters; Langmead and Salzberg, 2012) the reads to the mouse transcriptome (ENSEMBLE cDNA sequences or *ab initio* predicted genes, May 2014), the ERCC transcriptome and mapped reads counted (htseq-count; Anders et al., 2014) following the Cel-seq general pipeline.

### Transcriptome analysis

Mapped reads per cell were normalized as transcripts per million mapped reads (tpm) since cel-seq retains only one molecule per transcript (Hashimshony et al., 2012). For ERCC quantification experiments, shown in Figure S1 A+B, RT primers with unique molecular identifiers (UMI [6 nt] just before the barcode [6 nt]) were used to trace individual transcripts. Genes with tpm>5 were retained for further analysis which should reduce false positives from cross-contamination (see Methods section on cross-contamination) but also reduces low level expressed true detected genes by ~30%. Analysis was performed with cell groups in a pairwise fashion. Differentially expressed genes were assessed using single cell differential expression analysis (SCDE) using the R scde package (Kharchenko et al., 2014). Alternatively, we computed the absolute (*abs*) sigma-score (σ) for each gene by comparing mean and variance (*var*) of gene expression (*tpm*) of all cells between the 2 groups (*PopA, PopB*).

$$\text{sigma}-\text{score}(\sigma )=abs\left(\frac{mean\left(PopA\right)-mean\left(PopB\right)}{\sqrt{var{\left(PopA\right)}^{2}\;+var{\left(PopB\right)}^{2}}}\right)$$

Genes were ranked based on the sigma-score in a descending manner. Cells were shuffled between the two groups in a proportional and random fashion in order to randomize the dataset and sigma-scores were recalculated. We compared the Sigma-scores of the top 250 ranked genes between the original and shuffled groups. We statistically tested the two sigma-score distributions by Mann-Whitney *U*-test and retested by reshuffling multiple times. Statistical analysis of cell-types (PV-SOM, PV-VIP, VIP-SOM, L6-L2, CA1d-CA1v, L6Cal-L6Thal, L5Cal-L5Front) vs. shuffled distributions showed high significance levels (*P* < 10^−70^) while comparisons among shuffled groups showed much lower significance levels (1 > *P* > 10^−10^). Statistical analysis of cell groups (V1L2Ant-V1L2Post, PM-AL, V1->PM-V1->AL, HighSpeed-LowSpeed) vs. shuffled distributions and among shuffled distributions showed similar significance levels (1 > *P* > 10^−10^) indicating that cell groups (V1L2Ant-V1L2Post, PM-AL, V1->PM-V1->AL, HighSpeed-LowSpeed) had very similar gene expression (Figure S3).

Analysis was done using custom written scripts in python and VBA.

Correlation and cluster analyses were performed by first identifying genes with expression variability greater than noise using the provided code in R with standard parameters (CV = 50%, see also Figure S1; Brennecke et al., 2013) for each cell population comparison (e.g., PVCre vs. SOMCre, L2 vs. L6, PM vs. AL). The high variability identified genes were used for subsequent analyses. T-distributed stochastic neighbor embedding (t-SNE) analysis was performed using the Rtsne package (https://github.com/jkrijthe/Rtsne). Principal component analysis (PCA) was performed using the prcomp function. Pearson correlation was performed using Excel.

### (Cross)-contamination analysis

Cross-contamination can arise during library preparation when multiple single cell libraries are bulk processed as during the cel-seq protocol. We processed rat cortical pyramidal neurons, harvested identical to the mouse neurons, in parallel to the mouse neurons during library preparation. In order to estimate cross-contamination reads of rat and mouse cells were mapped to a modified rat mitochondrial genome. The rat mitochondrial genome was modified such that all sequence parts containing homologous identical sequences to the mouse mitochondrial genome were shorter than 40 bases, so that mouse mitochondrial reads (length 43 bases) could not map to the rat mitochondrial genome. Cross-contamination was assessed by first counting the reads mapped to the rat mitochondrial genome for each mouse cell and all rat cells (1 or 2) of the library. Cross-contamination per cell was then calculated as the fraction of mapped rat mitochondrial reads for each mouse cell of all mapped rat mitochondrial reads of the rat cell(s). Cross-contamination during library preparation was similar to published datasets (e.g., Macosko et al., 2015; Zeisel et al., 2015).

Contamination may also occur by accidentally harvesting surrounding tissue besides the cell of interest. Contamination was estimated by assessing GABAergic (GAD1, GAD2, Slc32a1) neuron and glutamatergic (Slc17a7) neuron specific transcript expression in glutamatergic and GABAergic neuron libraries, respectively.

GABAergic contamination estimate in glutamatergic neurons:

$$1-\frac{tpm(Slc17a7)}{tpm\left(Slc17a7\right)+mean\left(tpm\left(Gad1+Gad2+Slc32a1\right)\right)}$$

Glutamatergic contamination estimate in GABAergic neurons:

$$1-\frac{mean(tpm\left(Gad1+Gad2+Slc32a1\right)}{tpm\left(Slc17a7\right)+mean\left(tpm\left(Gad1+Gad2+Slc32a1\right)\right)}$$

Cell contamination was similar to published datasets (e.g., Zeisel et al., 2015; Cadwell et al., 2016; Tasic et al., 2016). Please note that cell contamination may also be the result of cross-contamination as described above or simply faint and varying coexpression of glutamatergic and GABAergic transcripts in single cells.

### Differential gene expression comparisons with published datasets

Gene expression comparisons were performed by comparing differentially expressed genes (SCDE) between our dataset and the Tasic et al. dataset (Tasic et al., 2016) for cortical GABAergic and glutamatergic neurons and the Cembrowski et al. dataset (Cembrowski et al., 2016a,b; http://hipposeq.janelia.org/) for hippocampal glutamatergic neurons. For the comparison of PVCre vs. SOMCre, SOMCre vs. VIPCre, PVCre vs. VIPCre, L2 vs. L6, L6Thal vs. L6Cal, and L5Cal vs. L5Front we matched the individual neurons to the molecular cell-types identified by Tasic et al. For this we built a classifier using marker genes identified by Tasic et al. as well as differentially expressed genes. Based on the classifier each neuron was assigned to a molecular cell-type defined by Tasic et al. We then selected a corresponding number of cells from the identified molecular cell-types (see Table S1) and performed SCDE analysis on the newly assembled datasets (e.g., PVTasic vs. SOMTasic, L2Tasic vs. L6Tasic). We compared SCDE identified differentially expressed genes from our and Tasic et al. datasets side by side (Table S1, normalized gene expression).

In order to compare hippocampal glutamatergic neurons (CA1d vs. CA1v) we took advantage of the Cembrowski et al. dataset. Differentially expressed genes from our dataset (SCDE) were directly compared side by side to differentially expressed genes identified by Cembrowski et al. using their gene enrichment analysis between glutamatergic neurons harvested from dorsal and ventral CA1. Due to differences in mouse genetic backgrounds, mouse age at sampling, sampling procedures (patch clamp harvest from acute slices vs. bulk dissected single cells/pooled cells) or library preparation our differential gene expression analysis allows for an approximate comparison with the published datasets. The dataset analyzed here was sequenced to depth of ~1.5 Mio transcriptome mapped reads. In comparison, Tasic et al. and Cembrowski et al. mapped ~2.5 Mio and >20 Mio reads to their transcriptomes.

### *In-vivo* imaging and photo-labeling of neurons

Craniotomy for imaging was performed as previously described (Lien and Scanziani, 2011). Briefly, animals (>p35, SepW1-Cre expressing Cre-dependent GCAMP6s/H2BpaGFP) were anesthetized using Isoflurane (0.5%) and Chlorprotixen (5 mg/kg, intraperitoneal) and urethane (1.2 g/kg, intraperitoneal). After skull exposure a metal head plate was fixed over the right visual cortex and a craniotomy (2.5 mm diameter) was performed, covered with agar and closed with a glass coverslip. After imaging the animals were prepared for slice preparation.

Two-photon imaging and photo-activation was performed in upper L2/3 (150–200 μm depth from pia) using a pulsed Ti:Sapphire laser (Chameleon Ultra II, Coherent) coupled to a Sutter Moveable Objective Microscope as previously described (Lien and Scanziani, 2011). SepW1-Cre mice expressing Cre-dependent GCAMP6s and H_2_BpaGFP were used to target superficial layer 2 neurons. Two-photon calcium imaging of GCAMP6s was performed at 950–1,000 nm and photo-activation of histone-bound photo-activateable GFP (H_2_BpaGFP) was done at 800 nm using ROIs (~10 × 10 μm) around the cell. All imaging and photo-activation was performed with Scanimage software. Calcium imaging of visual responses was performed at 2 frames/s.

### Visual stimuli and tuning analysis

Visual stimuli were generated in Matlab with Psychophysics Toolbox and displayed on an LCD monitor positioned 25 cm from the animal. Full contrast drifting square gratings (4 s) for high speed (0.025 cycles/deg spatial frequency; 4 Hz temporal frequency) and low speed (0.1 cycles/deg spatial frequency; 1 Hz temporal frequency) stimuli were presented at 8 different angles from 0 to 315° and a blank in randomized order for at least 8 times. Gratings were preceded and followed by a mean luminance gray screen (97 cd/m2) for 4 s. The 2 second mean luminance gray preceding each stimulus served as baseline. Fluorescent changes were calculated as dF/F averaged over at least 8 trials. The tuning of cells was analyzed by summing the averaged dF/F responses over all 8 directions. Cells were tuned (high speed or low speed) if the responses for one tuning stimulus were at least 4 times higher than for the other.

### Immunohistochemistry and cell quantification

Immunohistochemistry and cell quantification was performed as described previously (Pfeffer et al., 2013).

### *In-situ* hybridization comparisons

ISH images for selected genes were obtained from the Allen Institute mouse brain ISH database (Lein et al., 2007; http://mouse.brain-map.org/search/index). A representative part of primary visual cortex was used in comparisons. Comparisons were made by simple visual inspection.

## Author contributions

CP: Conceived the study and designed experiments with input from Massimo Scanziani; RB and CP: Performed all experiments except adult virus, beads; CTB injection performed by RB. Sequencing was performed at the UCSD Genome Center. CP: Wrote the manuscript with input from Massimo Scanziani and RB.

### Conflict of interest statement

The authors declare that the research was conducted in the absence of any commercial or financial relationships that could be construed as a potential conflict of interest.

## Supplementary material

Table S1 Data in this table are directly connected to supplementary figures S4, S5, S6, S8, S9, S12, S13. Each panel shows the dataset for the comparison of Pfeffer et al. vs Tasic et al. (S4, S5, S6, S8, S12, S13) or Cembrowski et al. (S9) as described in the text. The data are given as normalized (highest value per row and dataset set as 1) tpm values corresponding to the color coded heat map in each figure. Provided are also the log values of the differential expression significance as calculated by SCDE and sorted in a descending order. For each single cell the cell-type association and cell identity code are provided. CA1D vs CA1V comparison contains normalized values from the hipposeq dataset which are pooled cells (100 for each condition) in triplicate.
