# Supplementary material for: Correlating Anatomy and Function with Gene Expression in Individual Neurons by Combining in Vivo Labeling, Patch Clamp, and Single Cell RNA-seq
Source: Front Cell Neurosci. 2017 Nov 30;11:376. doi: 10.3389/fncel.2017.00376 (PMC5714881; doi:10.3389/fncel.2017.00376)
Supplement: Supplementary file 1 [file Image1.PDF]

Figure S1

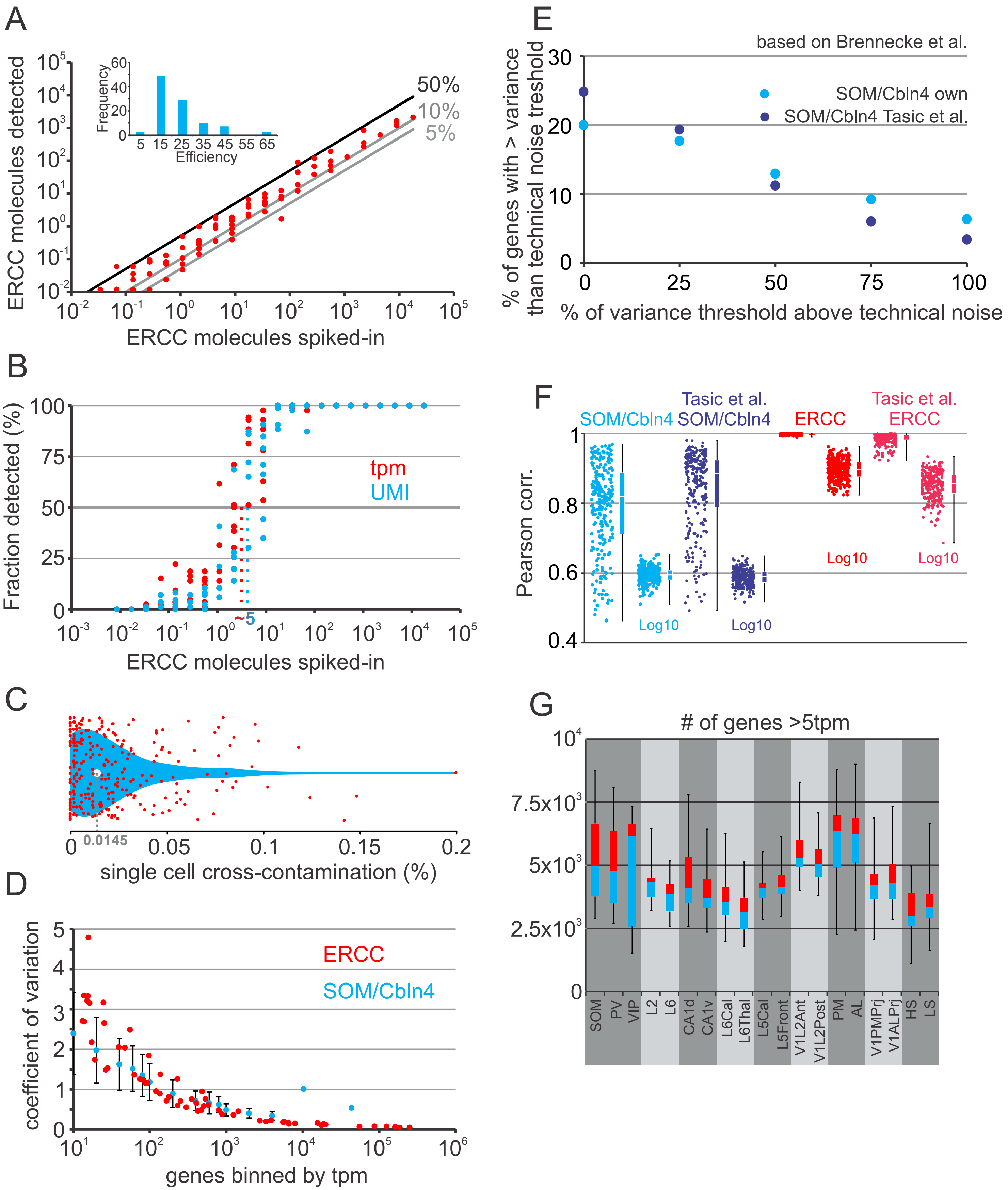
A
ERCC molecules detected
ERCC molecules spiked-in
Frequency
Efficiency
50%
10%
5%
B
Fraction detected (%)
ERCC molecules spiked-in
tpm
UMI
~5
C
0.0145
single cell cross-contamination (%)
D
coefficient of variation
genes binned by tpm
ERCC
SOM/Cbln4
E
based on Brennecke et al.
SOM/Cbln4 own
SOM/Cbln4 Tasic et al.
% of genes with > variance than technical noise treshold
% of variance threshold above technical noise
F
SOM/Cbln4
Tasic et al. SOM/Cbln4
ERCC
Tasic et al. ERCC
Pearson corr.
Log10
G
# of genes >5tpm
SOM
PV
VIP
L2
L6
CA1d
CA1v
L6Cal
L6Thal
L5Cal
L5Front
V1L2Ant
V1L2Post
PM
AL
V1PMPrj
V1ALPrj
HS
LS

Figure S1:
Efficiency, sensitivity, cross-contamination and variability of RNA-seq from patch-clamp harvested neurons.
Refers to results: RNA-seq of patch-clamped single neurons distinguishes major GABAergic cell-types
A) The quantity of ERCC spike-in control RNAs was analyzed after sequencing using unique molecular identifier primers. The number of spiked-in RNAs is plotted against the number of identified RNA molecules. The detection efficiency is about 10-15%. (n=88 ERCC samples)
B) The fraction of ERCC molecules that is detected is plotted against the number of spiked in molecules. 1 molecule of ~5 spiked in molecules is detected with 50% chance. Calculations are based on tpm (red) or UMI (blue).
C) Cross-contamination during library preparation. Cross-contamination was assessed by preparing libraries with 1 or 2 additional cells harvested from rat cortical slices. The number of reads of mitochondrial rat RNA was assessed and the percentage of cross-contamination is given for each mouse cell (blue points, see methods). The median cross-contamination is 0.0145%. Red plot shows a violin plot of the distribution.
D) Technical and harvesting/biological variability. The coefficient of variation was calculated for each gene of SOM/Cbln4 neurons (n=23, mean±stdev) and ERCC controls and plotted against the tpm. The genes of SOM/Cbln4 neurons were binned according to their tpm value while the ERCC genes were not binned. CV increases with decreasing expression level reflecting higher variability in gene detection for low-level expressed genes.
E) Gene expression variability above technical (ERCC) noise. Transcript expression variability was assessed using recent models (Brennecke et al., 2013) in comparison with the Tasic et al. dataset (Tasic et al., 2016). The percentage of genes above technical noise is plotted against the CV threshold above technical noise (0-100%) used for assessment. The variability of gene expression above technical noise is similar to the recently published Tasic et al. dataset on an overall level.
F) Scatterplots and boxplots of Pearson correlation coefficients of gene expression (tpm>5) between SOM/Cbln4 neurons (n=23 cells, n=253 pairwise correlations, light blue) and the respective ERCC controls (red). SOM/Cbln4: 0.79±0.126; SOM/Cbln4-log: 0.594±0.022;ERCC: 0.997±0.002; ERCC-log: 0.896±0.03;(mean±stdev). Pearson correlation coefficients are similar to a corresponding dataset of SOM/Cbln4 neurons of Tasic et al. (n=23 cells, n= 253 pairwise correlations, dark blue) and the respective ERCC controls (dark rosa). Tasic SOM/Cbln4: 0.839±0.115; Tasic SOM/Cbln4-log: 0.588±0.024;Tasic ERCC: 0.985±0.016; Tasic ERCC-log: 0.852±0.04;(mean±stdev).
G) Number of detected genes per sampled cell class for SOM (n=60), PV (n=71), VIP (n=43), L2 (n=17), L6 (n=17), CA1d (n=42), CA1v (n=37), L6Cal (n=53), L6Thal (n=32), L5Cal (n=29), L5Front (n=51), V1L2Ant (n=35), V1L2Post (n=39), PM (n=85), AL (n=78), V1PMProj (n=41), V1ALProj (n=42), HS - highspeed (n=40), LS - lowspeed (n=21) neurons shown as boxplot distribution.
