# Supplementary material for: Correlating Anatomy and Function with Gene Expression in Individual Neurons by Combining in Vivo Labeling, Patch Clamp, and Single Cell RNA-seq
Source: Front Cell Neurosci. 2017 Nov 30;11:376. doi: 10.3389/fncel.2017.00376 (PMC5714881; doi:10.3389/fncel.2017.00376)
Supplement: Supplementary file 2 [file Image2.PDF]

Figure S2

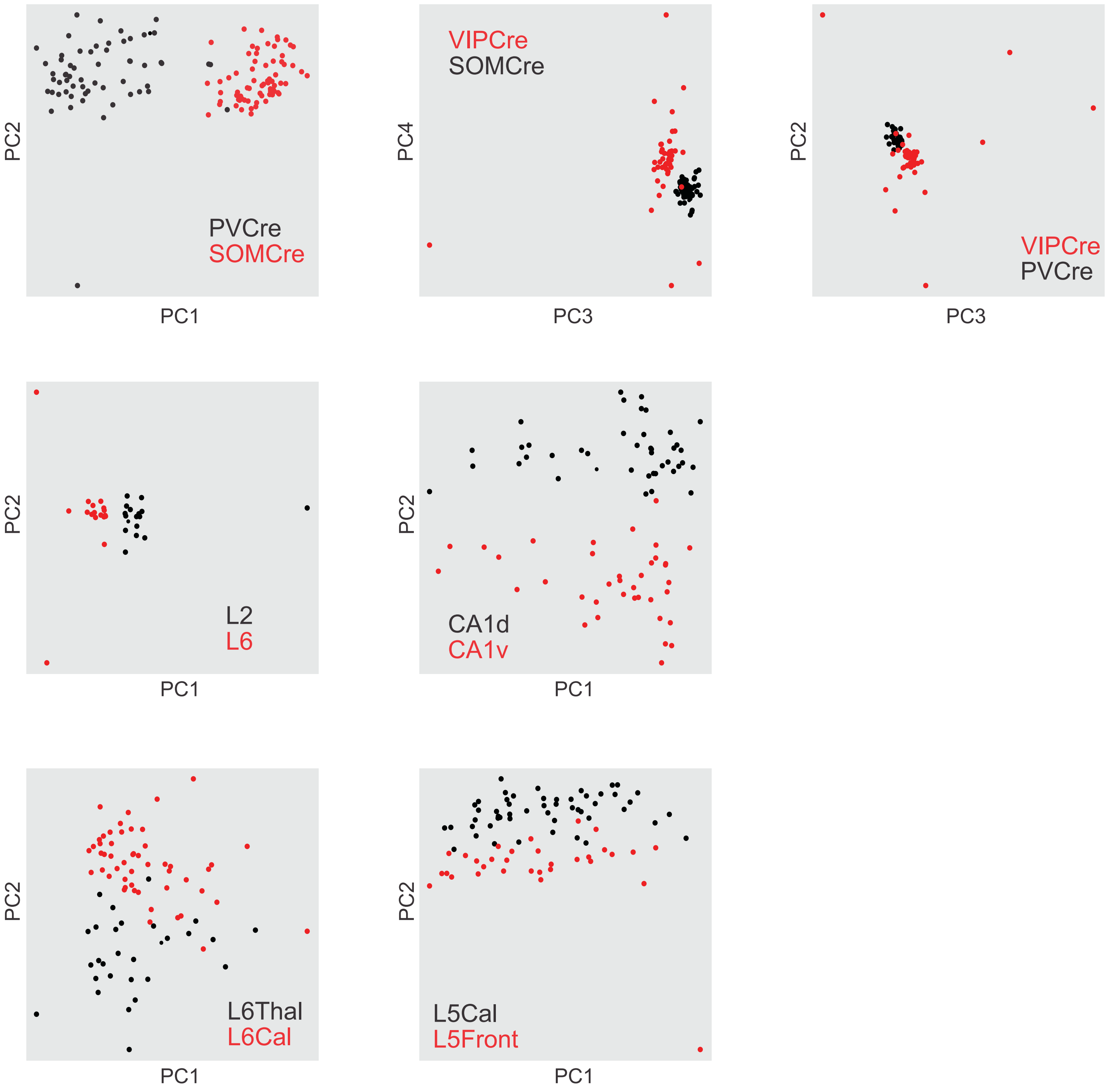

Figure S2:
Two-dimensional PCA representation of gene expression for cell population comparisons. Cells are color coded according to their cell population correspondance. SOMCre vs. VIPCre and PVCre vs. VIPCre showed best separation for PC3/4 and PC2/3, respectively.
