# Supplementary material for: Correlating Anatomy and Function with Gene Expression in Individual Neurons by Combining in Vivo Labeling, Patch Clamp, and Single Cell RNA-seq
Source: Front Cell Neurosci. 2017 Nov 30;11:376. doi: 10.3389/fncel.2017.00376 (PMC5714881; doi:10.3389/fncel.2017.00376)
Supplement: Supplementary file 3 [file Image3.PDF]

Figure S3

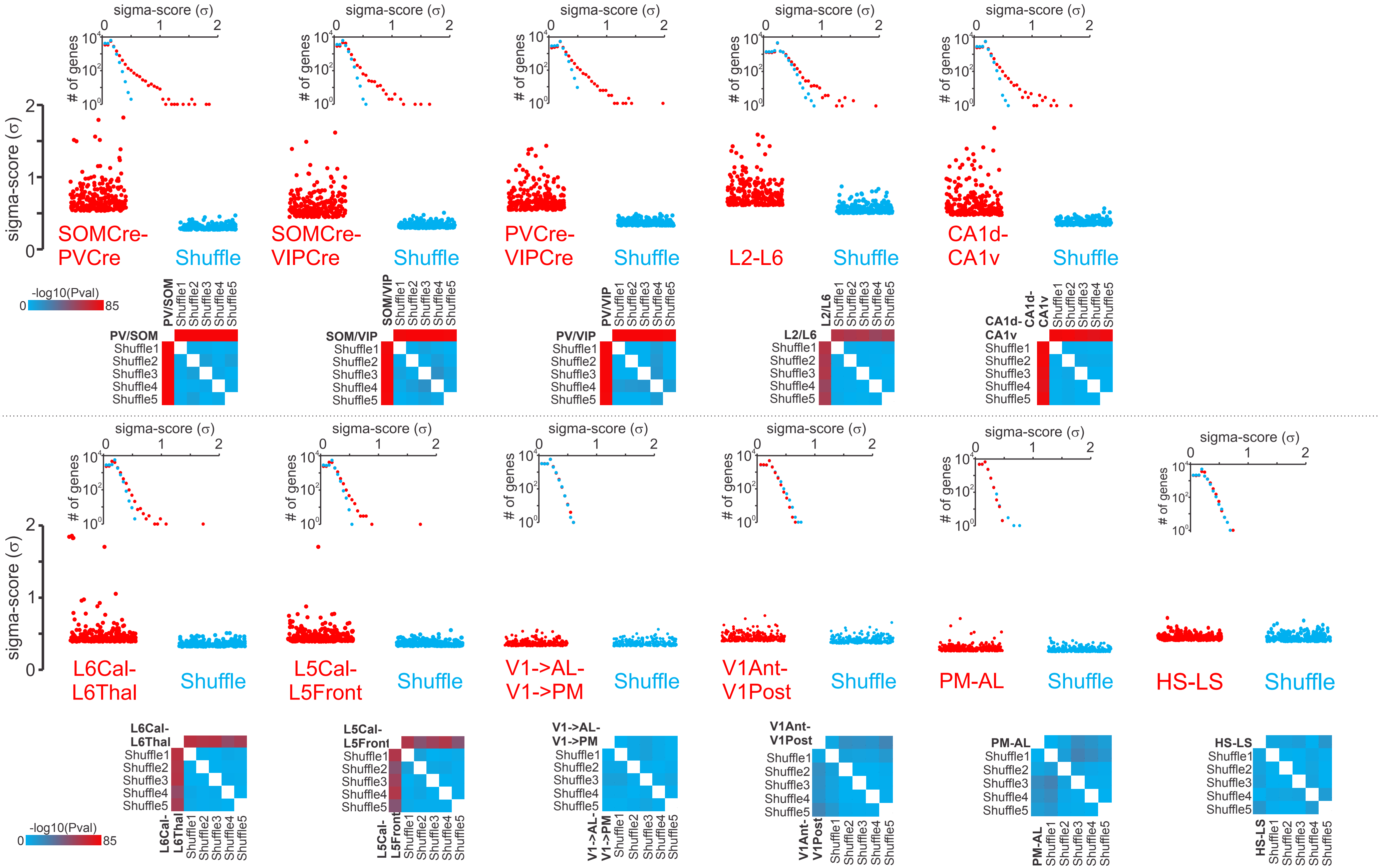

Figure S3:
Representation of sigma-scores for all cell population comparisons.
Sigma-scores were calculated for each gene in a pairwise comparison of cell populations (e.g. PVCre vs. SOMCre, L2 vs. L6). Genes were ranked for the highest sigma-score and the sigma-scores of the top 250 genes are plotted in a scatterplot for each pairwise comparison (red). Cells were shuffled equally between the groups maintaining total cell numbers per group and sigma-scores for each gene were recalculated and genes ranked accordingly (blue).
Insets above: Histogram showing number of genes plotted against the sigma-score for each pairwise comparison for the original (red) and shuffled (blue) cell distribution.
Insets below: Mann-Whitney U statistical analysis. Sigma-scores of top ranked 250 genes were statistically compared between cell populations and 5 different shuffled cell distributions and represented as a heat map.
