# Supplementary material for: Correlating Anatomy and Function with Gene Expression in Individual Neurons by Combining in Vivo Labeling, Patch Clamp, and Single Cell RNA-seq
Source: Front Cell Neurosci. 2017 Nov 30;11:376. doi: 10.3389/fncel.2017.00376 (PMC5714881; doi:10.3389/fncel.2017.00376)
Supplement: Supplementary file 4 [file Image4.PDF]

Figure S4

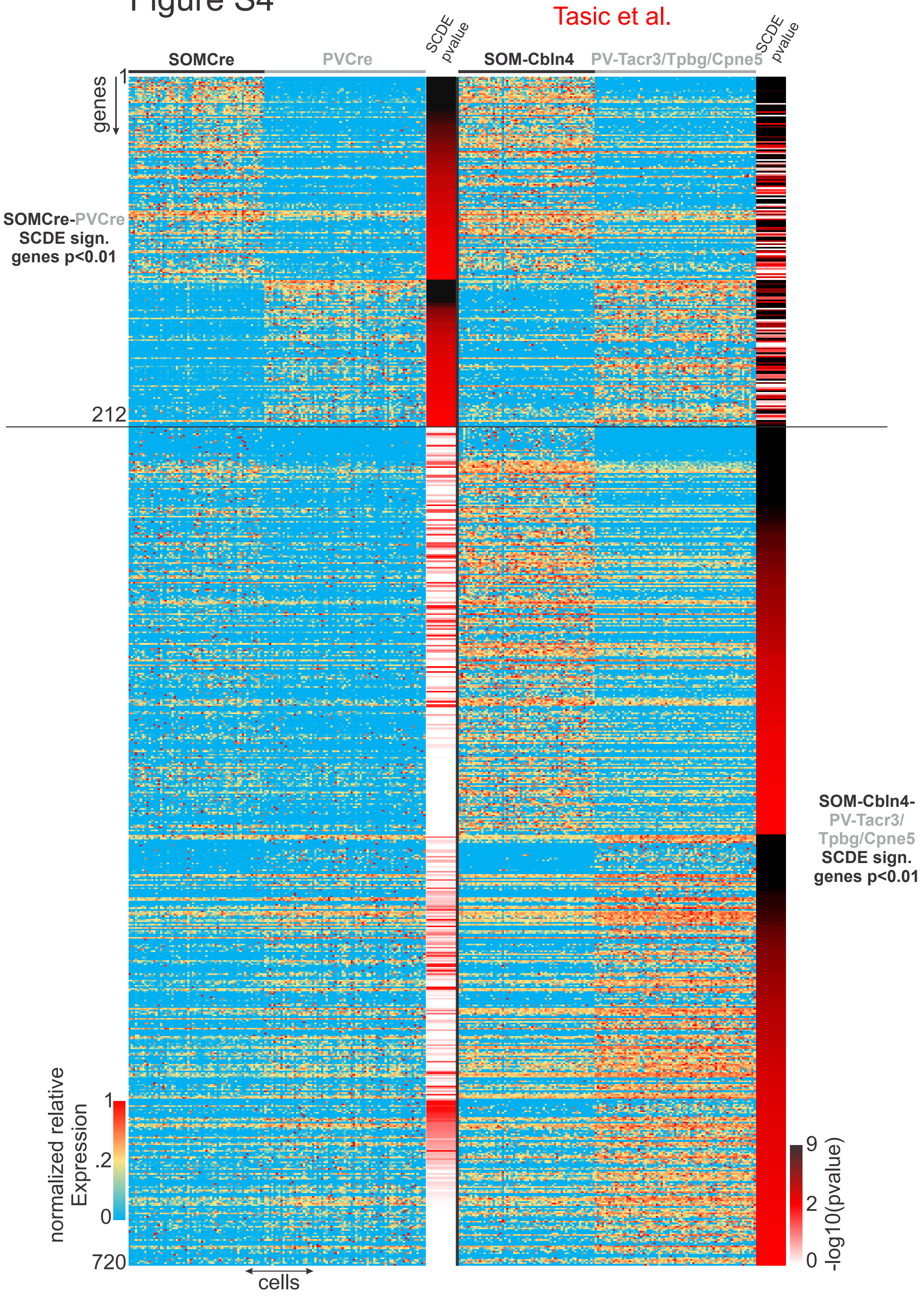

Figure S4: Comparison of datasets: PVCre/SOMCre vs. Tasic PV/SOM

Cells from this dataset were classified and matched to the molecular cell-types identified by Tasic et al. and corresponding cells were selected from the Tasic et al. dataset. SOMCre cells (harvested in L2/3) most closely matched Tasic et al. SOM/Cbln4 cells, with the exception of 3 neurons which identified as PV. For simplicity all selected Tasic et al. cells belonged to SOM/Cbln4 molecular cell-type. PVCre cells most closely matched PV-Tacr3, PV-Tpbg, and PV-Cpne5 cells.Differentially expressed genes were identified using SCDE for the dataset from this study and the Tasic et al. dataset (Tasic et al. 2016). Genes were sorted according to expression selectivity and p-value of differential expression (color coded on the right of each dataset). Data are presented in columns (cells) and rows (genes) and color coded according to the normalized relative expression level for each gene (color code on the left bottom). Top 212 genes (until horizontal black line) are genes identified as differentially expressed from this study (p<0.01) and bottom genes (below black horizontal line) identified from the Tasic et al. dataset. Due to space restraints gene names are not given (listed in Table S1).
