# Supplementary material for: Correlating Anatomy and Function with Gene Expression in Individual Neurons by Combining in Vivo Labeling, Patch Clamp, and Single Cell RNA-seq
Source: Front Cell Neurosci. 2017 Nov 30;11:376. doi: 10.3389/fncel.2017.00376 (PMC5714881; doi:10.3389/fncel.2017.00376)
Supplement: Supplementary file 7 [file Image7.PDF]

Figure S7

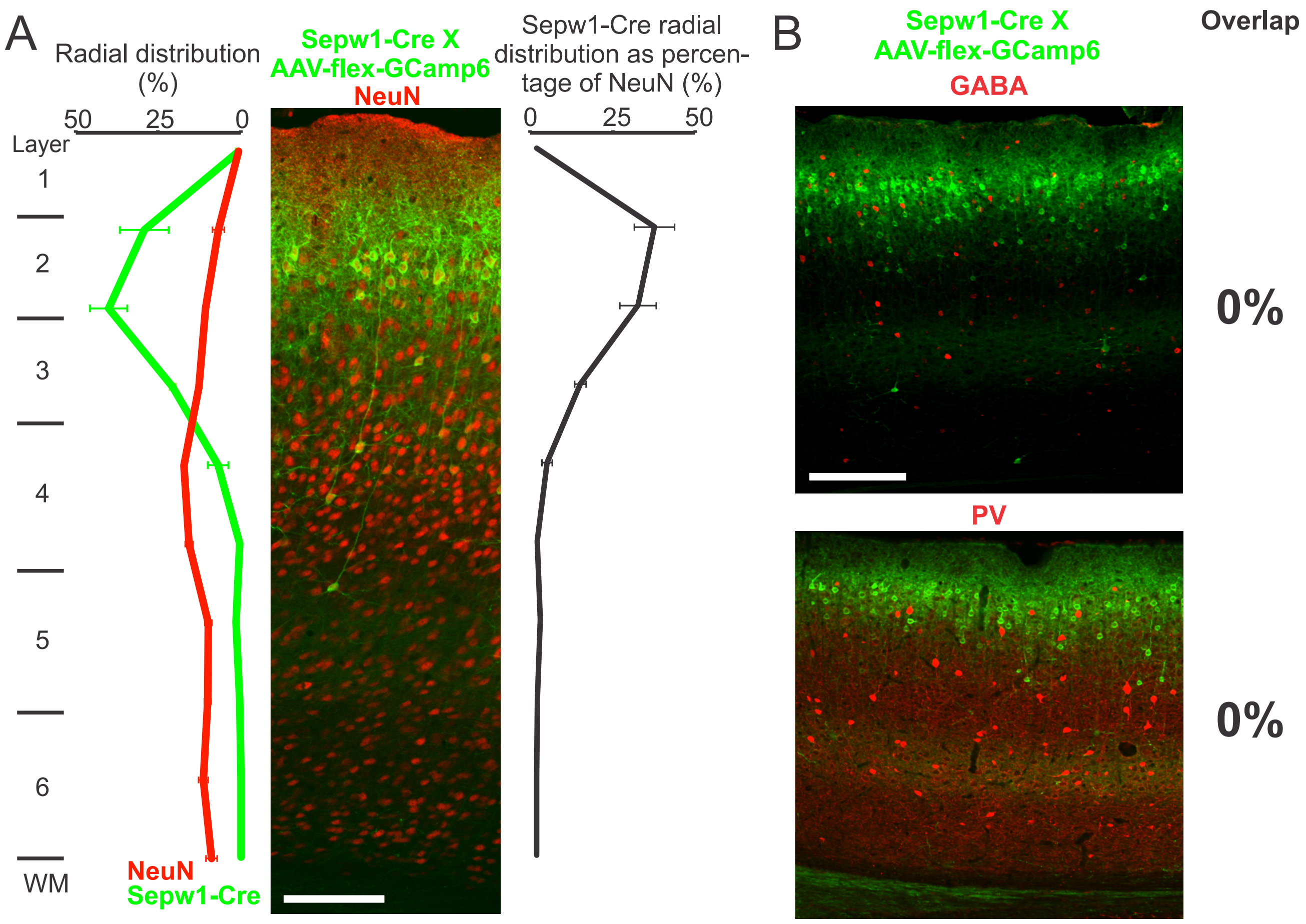

Figure S7:

Distribution and overlap with GABAergic markers of SepW1-Cre positive neurons in mouse primary visual cortex. Refers to Figure 2,5.
A) Immunostaining for GFP of GCamP6s transfected neurons of mouse primary visual cortex shows labeling in superficial layer 2 that is quantified along with the general neuronal marker NeuN. Scalebar: 100µm
B) Co-staining for GFP in SepW1-Cre transfected neurons and GABAergic markers GABA and PV show no overlap in primary visual cortex indicating that SepW1-Cre neurons are glutamatergic. Scalebar: 200µm
