# Supplementary material for: Correlating Anatomy and Function with Gene Expression in Individual Neurons by Combining in Vivo Labeling, Patch Clamp, and Single Cell RNA-seq
Source: Front Cell Neurosci. 2017 Nov 30;11:376. doi: 10.3389/fncel.2017.00376 (PMC5714881; doi:10.3389/fncel.2017.00376)
Supplement: Supplementary file 8 [file Image8.PDF]

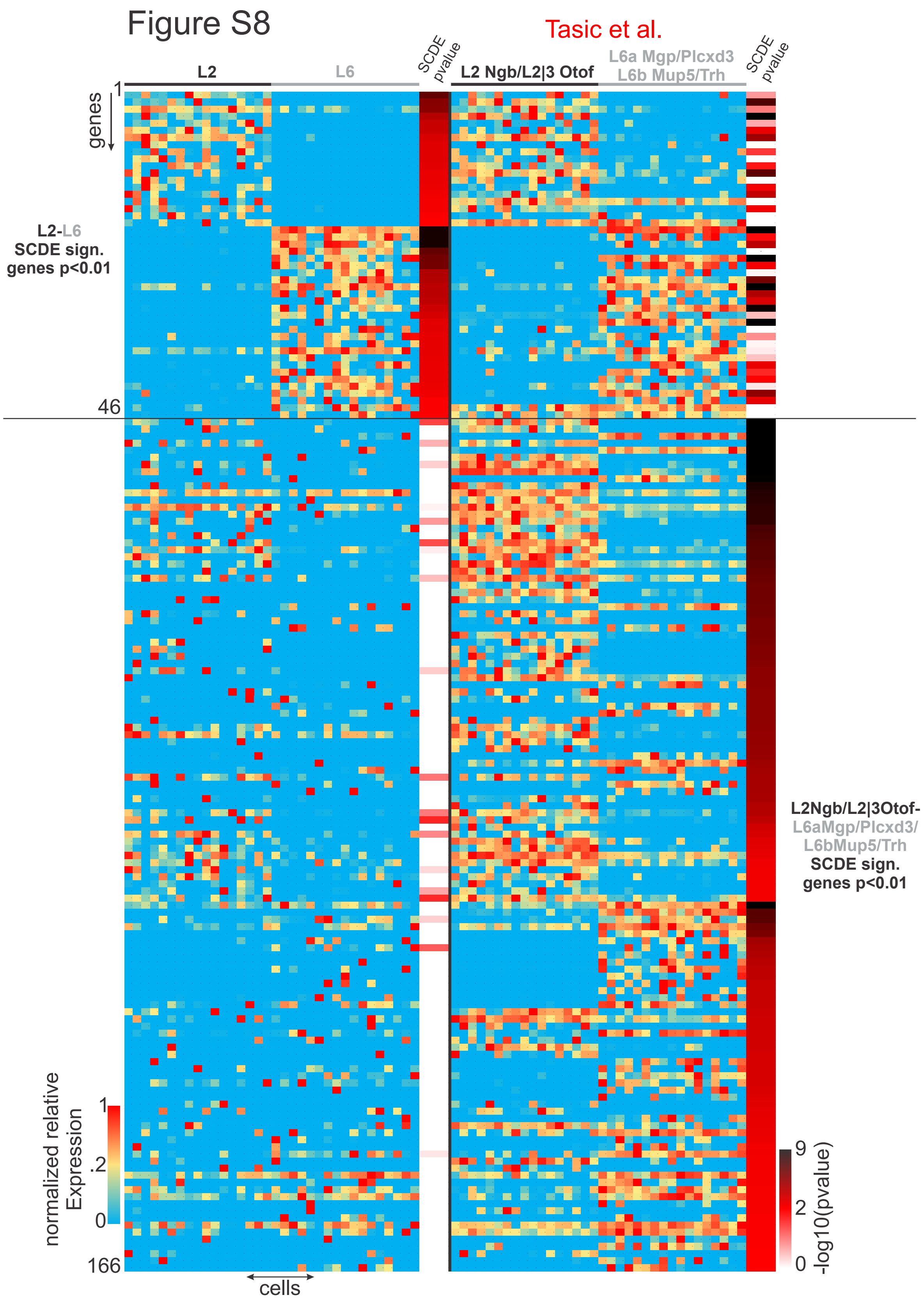

Figure S8: Comparison of differentially expressed genes between datasets: L2/L6 vs. Tasic L2/L6
Cells from this dataset were classified and matched to the molecular cell-types identified by Tasic et al. and corresponding cells were selected from the Tasic et al. dataset. L2 cells most closely matched L2-Ngb and L2/3-Otof cells. L6 cells most closely matched L6a-Mgp, L6a-Plcxd3, L6b-Mup5, and L6b-Trh cells. Differentially expressed genes were identified using SCDE for the dataset from this study and the Tasic et al. dataset (Tasic et al. 2016). Genes were sorted according to expression selectivity and p-value of differential expression (color coded on the right of each dataset). Data are presented in columns (cells) and rows (genes) and color coded according to the normalized relative expression level for each gene (color code on the left bottom). Top 46 genes (until horizontal black line) are genes identified as differentially expressed from this study ($p<0.01$) and bottom genes (below black horizontal line) identified from the Tasic et al. dataset. Due to space restraints gene names are not given (listed in Table S1).
