# Supplementary material for: Correlating Anatomy and Function with Gene Expression in Individual Neurons by Combining in Vivo Labeling, Patch Clamp, and Single Cell RNA-seq
Source: Front Cell Neurosci. 2017 Nov 30;11:376. doi: 10.3389/fncel.2017.00376 (PMC5714881; doi:10.3389/fncel.2017.00376)
Supplement: Supplementary file 9 [file Image9.PDF]

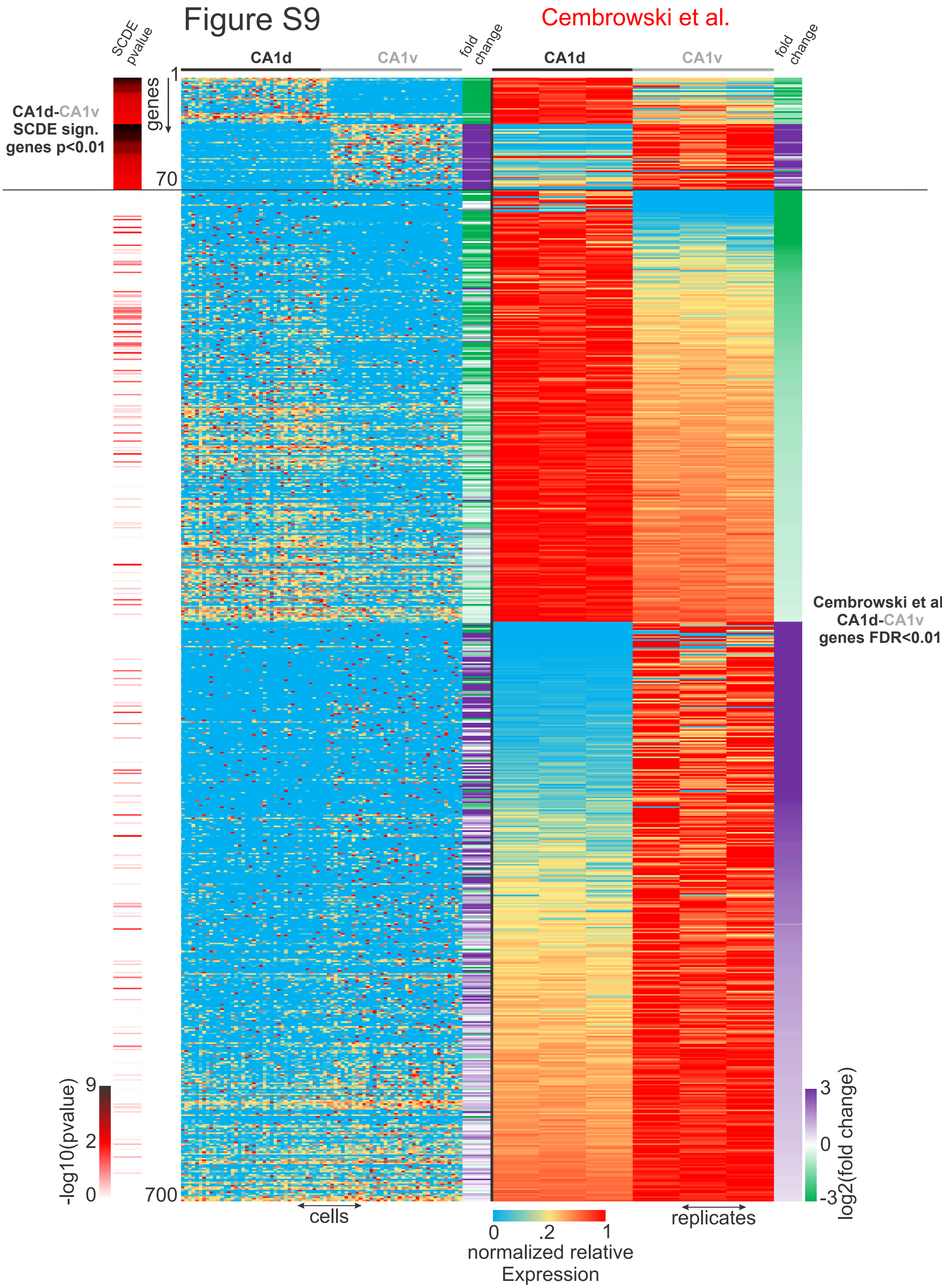

Figure S9: Comparison of differentially expressed genes between datasets: CA1d/CA1v vs. Cembrowski CA1d/CA1v
Cells from this dataset were directly compared to the CA1d/CA1v dataset by Cembrowski et al. Differentially expressed genes were identified using SCDE for the dataset from this study and directly obtained from the Cembrowski et al. dataset (Cembrowski et al. 2016a,b). Genes were sorted according to expression selectivity and p-value of differential expression for this dataset (top genes, color coded on the left) and for the Cembrowski et al. dataset according to the log2(fold change) (color coded on the right of each dataset). Data are presented in columns (cells) and rows (genes) and color coded according to the normalized relative expression level for each gene (color code given below). Top 70 genes (until horizontal black line) are genes identified as differentially expressed from this study (p<0.01) and bottom genes (below black horizontal line) identified from the Cembrowski et al. dataset. Due to space restraints gene names are not given (listed in Table S1). Please note that Cembrowski et al. used gene enrichment analysis by processing triplicates of ~100 selected and pooled neurons from the dorsal and ventral pole of CA1.
