# Supplementary material for: Correlating Anatomy and Function with Gene Expression in Individual Neurons by Combining in Vivo Labeling, Patch Clamp, and Single Cell RNA-seq
Source: Front Cell Neurosci. 2017 Nov 30;11:376. doi: 10.3389/fncel.2017.00376 (PMC5714881; doi:10.3389/fncel.2017.00376)
Supplement: Supplementary file 10 [file Image10.PDF]

Figure S10

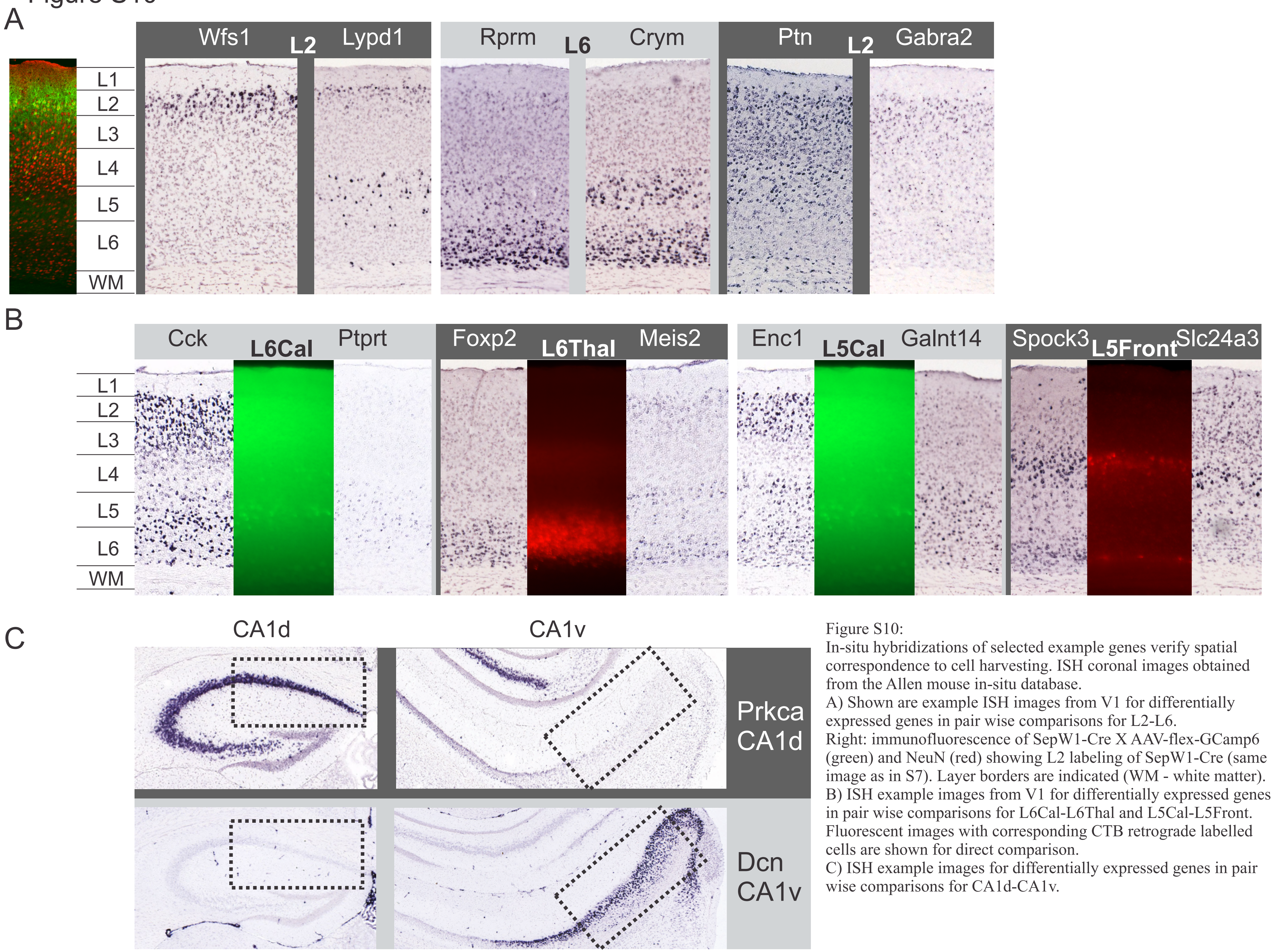

Figure S10:
In-situ hybridizations of selected example genes verify spatial correspondence to cell harvesting. ISH coronal images obtained from the Allen mouse in-situ database.
A) Shown are example ISH images from V1 for differentially expressed genes in pair wise comparisons for L2-L6.
Right: immunofluorescence of SepW1-Cre X AAV-flex-GCamp6 (green) and NeuN (red) showing L2 labeling of SepW1-Cre (same image as in S7). Layer borders are indicated (WM - white matter).
B) ISH example images from V1 for differentially expressed genes in pair wise comparisons for L6Cal-L6Thal and L5Cal-L5Front. Fluorescent images with corresponding CTB retrograde labelled cells are shown for direct comparison.
C) ISH example images for differentially expressed genes in pair wise comparisons for CA1d-CA1v.
