# Supplementary figures and images for: Correlating Anatomy and Function with Gene Expression in Individual Neurons by Combining in Vivo Labeling, Patch Clamp, and Single Cell RNA-seq
Source: Front Cell Neurosci. 2017 Nov 30;11:376. doi: 10.3389/fncel.2017.00376 (PMC5714881; doi:10.3389/fncel.2017.00376)

# Figure S11

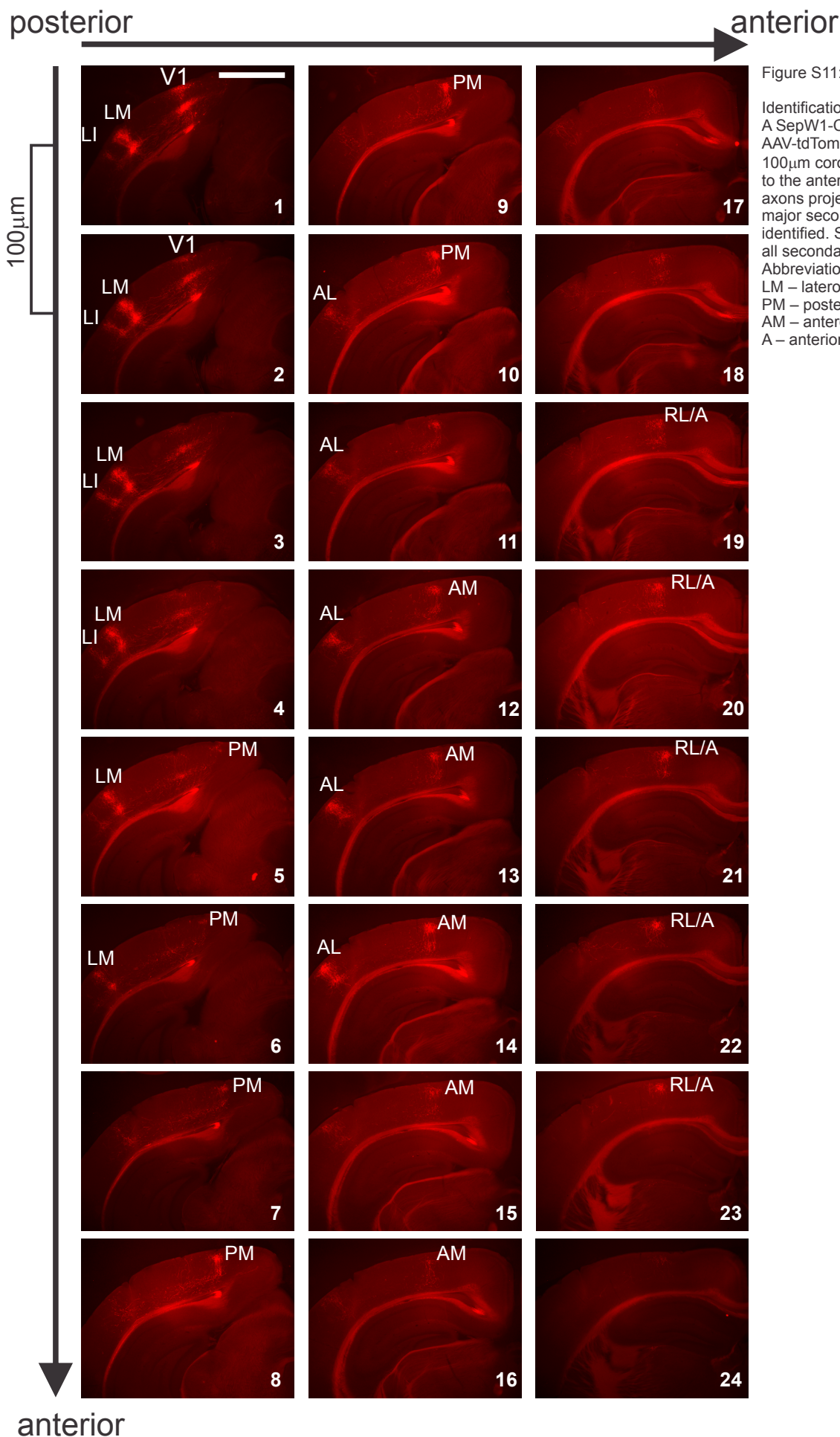

Supplement: Supplementary file 11 [file Image11.PDF]
